# On Insanity and Lunatic Asylums in Norway

**Published:** 1858-04-01

**Authors:** W. Lauder Lindsay

**Affiliations:** Physician-Superintendent of Murray's Royal Institution for the Insane, Perth


### Art. III.—ON INSANITY AND LUNATIC ASYLUMS IN NORWAY.

BY W. LAUDER LINDSAY, M.D., F.L.S., ETC.,

*Physician-Superintendent of Murray's Royal Institution for the Insane, Perth.*

A HOLIDAY tour in Norway during August last (1857) afforded me an opportunity of visiting the best Norwegian asylums, and of meeting and conversing with the first authorities on Insanity, the Lunacy Laws, the Treatment of the Insane, and the Construction and Management of Asylums, in that country. From the fact that few of our British psychologists, or physicians generally, have visited, or are in the habit of visiting, Norway, and that still fewer possess a knowledge of the Norwegian language and literature, much less is known in this country of the insane and asylums of Norway than of most other European countries. Hence we are apt to suppose that Norway—that Scandinavia generally—

is behind us—behind the age—in its fiscal and medical treatment of its insane. But such is very far from being the case. Norway has reason to be proud of her liberal and enlightened treatment of her insane; and we in Britain may learn not a few useful lessons by the study of her Lunacy Laws, and of her modern asylums. The history of the treatment of the insane in Norway is somewhat similar to that in our own country. There was a period of cloud and darkness—of ignorance and prejudice—of restraint and brutality: then followed a revolution—in Norway dating from the Law of the 17th of August, 1848; and now the sunshine has burst forth, with all its vivifying influences, and liberality, and enlightenment, science and humanity are introducing the "moral system" and the law of kindness to the fullest extent. The State Asylum of Norway, at Christiania, is but a very few years old: it is worthy of the sagacity and liberality of the Norwegians. It embodies all the most recent Continental views on the management of the insane and the construction of asylums; indeed, it may be regarded as a reflection, or concentration, of the excellencies of the German asylums especially. These facts or opinions lead me to believe that some account of the treatment of insanity, and the construction and management of asylums, in Norway, will not be unacceptable to British psychologists. I shall first give a short history relative to the State Asylum for Norway, and the City Asylum proper, both in Christiania; and shall thereafter make a few remarks on the Norwegian Lunacy Laws, and on the statistics of insanity in Norway.

In describing the State Asylum at Gaustad, Christiania, I must draw largely on the first Annual Report of the institution, admirably drawn up by Dr. Sandberg, the Physician-Superintendent.* It is a quarto pamphlet, of forty-three pages, with numerous schedules and tables, and with plans of both floors of the asylum. Being a first Annual Report, it is unusually full, containing a most interesting account of the history of the institution, its cost, the architectural and economical details, as well as the ordinary routine of an Asylum Report. It consists of two sections; of these, the first is divided into the following subsections or chapters:—*a.* Site; *b.* Ground-plan of building; *c.* Water supply; *d.* Drains and sewers; *e.* Gas supply; *f.* Pleasure-grounds and garden: while the second contains chapters on—*a.* Dietary; *b.* Residence of patients; *c.* Forms of disease; *d.* Medical treatment; and *e.* Terminations of disease. The documents appended to the Report consist of a ground-plan,

* "Generalberetning fra Gaustad Sindssygeasyl, for Aaret 1856, ved Ole Sandberg, Direktör. Christiania: Trykt i det steenske Bogtrykkeri. 1857."
"General Report of the Gaustad Lunatic Asylum, for the Year 1856, by Ole Sandberg, Physician-Superintendent." (It bears date February, 1857.)

and plan of the second floor of the institution; statistics regarding the admissions, discharges, and deaths; amount and value of work done by patients; schedule of forms of restraint; and a full table of the new dietary regulations. I have sufficiently indicated the nature of the contents of the Report: to its pages I must refer the reader for fuller details than I am able to give here regarding the Norwegian State Asylum.

The State Asylum is an imposing palatial building, perched conspicuously on an eminence at Gaustad, about three and a half miles north-west of the town of Christiania, its site being one of the finest parts of Akersdal. It stands at a considerably higher elevation than Christiania. Its exposure is to the south; and it commands a magnificent prospect down the beautiful Christiania Fjord. It could scarcely possess a more beautiful site: the view surpasses that from any British asylum with which I am acquainted. So prominent is its position, that it is one of the first objects to arrest the attention of the traveller or tourist who approaches Christiania by sea; and on this account it is generally noticed in works descriptive of Norwegian topography—works which are now neither few nor far between, in consequence of the numbers of tourists, possessed of the *cacoëthes scribendi,* who annually visit fair Norway. The building is evidently quite new: the airing-courts and pleasure-grounds are only yet in process of being properly laid out. The building itself is not yet complete. I had the good fortune to be present at a meeting of the "Storthing," or Norwegian Parliament, when a vote of 26,000 specie dollars* was granted for the completion of the original design. The worthy Physician-Superintendent, Dr. Sandberg, was present during the discussion, and evidently took a very warm interest in the issue of the proceedings. In proportion to its accommodation, this institution appears, on first sight, a very costly one. It has already cost—for erecting the edifice and constituting the establishment—upwards of 250,000 specie dollars; and, when finished, it will have cost upwards of 300,000 —equal to about 70,000*l.*! This gives a present expense per patient of 1200 to 1300 specie dollars, or between 250*l.* and 300*l.*; and a prospective expense of 1400 specie dollars, equal to more than 300*l.* a-head. Some English asylums have certainly cost sums as large, or nearly so—from 200*l.* to 300*l.* per head. But that such a heavy expenditure is not absolutely necessary is shown by the example of the new Montrose Royal Asylum—which is allowed, by competent judges, to be a model institution, so far as architectural arrangements are concerned, for the pauper insane—and which is now being erected at a total cost of 50*l.* per

* The Norwegian specie dollar is equal to about 4*s.* 6*d.* English; or, in other words, 1*l.* English is equal to about four and a half Norwegian dollars.

head, or 14,000*l.* for about 300 patients. The disproportion between this cost and that of the Gaustad Asylum of Christiania is very striking; for, at present, the latter accommodates only about 160 or 170 patients, and, when completed, will not hold more than 250! In establishing a State Asylum which should be a model for the whole country—a model from which future minor asylums, in different parts of the country, will be built—the Norwegians resolved—and, to a certain extent, rightly resolved—to spare no expense in bringing the asylum up to the requirements of the age—in introducing all the most modern improvements—in supplying a full and efficient staff. But it admits of a reasonable degree of doubt, whether they have not paid too dear for their whistle; and whether there has not been, for instance, in regard to the arrangement of the wings of the building, a sacrifice of real usefulness to theoretical advantages and imposing display. The institution is probably on too extensive a scale for 250 patients: its area is too expanded, causing a dissipation of time and energy on the part of the officers. But the means of classifying the inmates—of separating the noisy and dirty from the quiet and orderly—are undoubtedly remarkably good. In building their asylum, the cure and comfort of the patients has evidently been the ruling and guiding idea; the expense has been a subsidiary—a secondary question. Everything has been done to render the establishment complete as an hospital for the treatment of insanity; everything introduced has been introduced with a view to cure, mediately or indirectly—site, view, work, amusement, furniture, &c. It may be useful, though not a little humiliating, to contrast this tendency—this desire—this unselfish liberality and enlightenment—with the theory, or at least the practice, of certain ratepayers at home, who build asylums, like workhouses, in the cheapest possible way, and without the remotest idea of, or reference to, cure, or perhaps even comfort. A disagreeable burden is cast upon their shoulders—that of maintaining their insane fellow-creatures; and they ease themselves thereof in a way which may be satisfactory to their pockets, but can scarcely be so to their consciences—to their sense of duty and justice. It is right to state here that some of the architectural arrangements of Gaustad Asylum do not meet with the approval of the Superintendent, Dr. Sandberg, who appeared to me a most intelligent, practical man, of the German school of psychologists. I know not to what extent medical men conversant with the requirements of asylums were consulted in the construction of this establishment; but from what I know of the Norwegians generally, and of their mode of transacting business, I have no doubt that their State Asylum is the result of a careful study of the best models on the Continent of Europe, by architects and medical *experts* conjointly. This is but as it should be: if there be faults in construction, they arise not from ignorance, but from errors in judgment. Had such a policy been pursued in certain British asylums, we should not now have to deplore some gigantic, expensive, and absurd blunders. There is, unfortunately, in regard to the construction of asylums, too frequently a tendency to trust everything to architects, who have never practically felt, nor seen, the requirements of an hospital for the insane, and who are naturally more apt to be guided by questions of finance—by matters of mere appearance, or apparent neatness—than by the adaptation of means to an end—that end being cure and comfort of patients, not £ *s. d.* A medical superintendent has nothing to do with the designing or erecting of the building; he is not appointed till the hospital is about to be opened—in time, in fact, only to discover its imperfections without having it in his power to remedy them. This subject is one of great importance to Scotland at a time when the Scotch counties are about to build a series of district pauper asylums, similar to those of England and Ireland. I have no hesitation in affirming that the first step in establishing an asylum should be the appointment of a medical superintendent, who ought to be consulted as to site, expense, mode of constructing, heating, ventilating, water supply, laying out of grounds, &c. The money expended in his salary during a year or a couple of years prior to the opening of the institution would, I feel assured, be ultimately and fully saved in the value of his advice and superintendence, and in the better adaptability of the establishment to the purposes for which it was founded. Most of the older Scotch asylums, which were designed and built entirely by architects, labour under irremediable defects in construction; and only last summer, in Ireland, in some new asylums, or parts of asylums, built by Government, I saw several most expensive and grievous errors, which arose out of the simple fact that no medical *expert* had ever been consulted regarding such asylums, or departments thereof. Before leaving this subject, I may state that I found the Norwegians, generally, complaining of the expensiveness of Gaustad; and there were serious apprehensions that the Storthing would not give a grant of the 26,000 specie dollars necessary for the completion of the present buildings. Hence, doubtless, the anxiety, and the presence, of Dr. Sandberg at the debate on the subject.

The building consists of four parallel wings, separated from each other by commodious airing courts, and communicating by covered archways and passages. They are also divided laterally into two symmetrical series—male and female sides, or departments—by a central space, which contains the "Ökonomibygning," or offices, &c. These wings are built somewhat alike, with the exception of the fourth, or back wing, which has at right angles to it a supplementary wing, devoted to dirty cases. They are called respectively A, B, C, D, and E, and are set apart for the following classes or kinds of patients :—A, for quiet patients of the better ranks of life—for the inmates consist of an admixture of pauper and private patients, precisely as in most of the existing Scotch asylums; B, for quiet and orderly patients of the pauper class; C, for the noisy and turbulent; D, for the violent, excited, and destructive; and E, for the dirty and degraded. All the wings are of two stories, with the exception of the fourth, or D, E, which, along with the porter's lodge, laundry, and kitchen, consists of one only. The free, or outer, end of each of the three front wings, A, B, and C, terminates in a couple of four-sided compartments, having externally buttressed projections, with windows resembling bow-windows; the one forms part of the sitting-room, work-room, or parlour; the other is occupied by the water-closet. The front wing, A, facing the south, and commanding the finest views, is devoted to quiet patients of the higher classes (*afdelingen for de rolige Syge af den dannede Klasse*). The ground floor contains, at the outer end of the corridor or gallery, the parlour or association-room (*samlingsværelse*),—a large, cheerful, comfortable room, handsomely furnished, and commanding magnificent views over the country intervening between Gaustad and Christiania, of the town of Christiania, and down the Christiania Fjord. The furniture is quite of the style of ordinary drawing-room or parlour furniture, including handsome ottomans; while pictures deck the walls, flowers ornament the tables, and books and newspapers are strewed about everywhere. The furniture is tasteful and modern; the painting or papering of the walls is new and fresh; the rooms have much of the aspect and feeling of home; and, indeed, everything at present appears to the greatest advantage. Ten or twenty years will work a change in the character of Gaustad, in all probability: the architectural disadvantages will have fully appeared; the building will look somewhat old; the officers may be less enthusiastic and full of vigour; the furniture will be the "worse of the wear;" and the Norwegians may have learned to build less expensive and imposing asylums! On the same floor are two single bed-rooms (*enkeltværelser*), and five double-rooms or dormitories for two patients (*dobbeltværelser*). There is only one water-closet, and that one far from commodious. The corridor or gallery is too narrow—very different from the spacious, handsome, light, airy corridors of some of the recently built English asylums. On the second, or higher story, there are a large association-room (*forsamlings-sal*),* with a finer and more extensive view than that from the corresponding

* I do not know what distinction is drawn between "samlingsværelse" and "forsamlings-sal," unless the former is an ordinary parlour, and the latter more properly an assembly-room.

room on the ground floor, five single bed-rooms, three double bed-rooms, and an attendant's room (*tjenerværelse*). The second wing, B, is occupied by quiet patients of the poor or pauper class (*afdelingen for rolige Syge af Almues klassen*). The ground floor contains, at the near end of the gallery, a dining-saloon (*spiseværelse*), three dormitories (*soveværelser*), and a work-room at the further end of the gallery, in the place occupied by the sitting-room or parlour in the front wing, A, and of the same size and form. The corridor and water-closet are of the same size as in front wing. The second story contains a large association-room, of the same size and position as in front wing, and five dormitories. The third wing, C, is set apart for the turbulent and noisy (*afdelingen for urolige Syge*). The ground floor contains a large work and day-room at free end of gallery, of same size as work-room in wing B, nine single bed-rooms, and corridor and water-closet, as in last wing, B. The second story possesses four dormitories (*fælles Soveværelse*), one single bed-room, and an association-room, at farther end of gallery, of same size and position as in two front wings. The excited and destructive cases occupy the fourth, or back wing; and the dirty, the sub-wing at right angles thereto, but connected therewith (*afdelingen for rasende og for urenlige Syge*). There are here an association-room (*forsamlingsværelse*), at the angle of junction of the two wings, two watch-rooms or attendants' rooms (*vogterværelser*), a bath-room, water-closet, drying-room (*törreværelse*), and thirteen "cells" (*celler*) or single rooms, lit from above.

Between the male and female sides of the third wing is the laundry (*Vadskeri*); and between the two halves of the second wing stand the offices, known collectively as the "Ökonomibygning." This consists of two stories, and includes, on the lower or ground floor, the offices of the superintendent and steward, the medical library and consulting-room, the visitors' room, the steward's residence, kitchen, larder, pantry, laundry, store-rooms, and servants' rooms; and, on the second story, the chapel and recreation-room, chaplain's residence, and apartments belonging to medical assistant, apothecary, housekeeper, and other officers. These offices, &c., are connected directly by a gallery or passage with the male and female sides of the second wing. The house-steward's office is a roomy, comfortable, counting-room, in which several clerks were busied with the house books. There is a great want of this department, and of such an officer, in many of our British asylums. In too many cases is all, or great part, of the steward's work devolved on the unfortunate superintendent, who becomes, or is made to become, a mere drudge, a "Jack-of-all-trades," and who cannot possibly properly discharge the medical duties of his office when fagged and exhausted by the merest clerk's work—by the attention necessary to the keeping in order several dozens of ponderous and absurd books. The medical superintendent has here also a handsome and commodious parlour adjoining the steward's office, as a consulting-room, where he can conveniently confer with his subordinates or with visitors. This room contains a good medical library, supplied by the asylum—a library embracing works, and all the periodicals, on insanity, in every European language. I noticed the "Journal of Psychological Medicine," occupying a prominent place on its shelves, besides a variety of English works on insanity. The establishment of such a library, for the benefit of the medical and other officers, is worthy of imitation in this country. In too many cases the superintendent cannot afford to purchase expensive foreign works on insanity; and directors of asylums seem either conveniently ignorant that there is such a thing as a literature of insanity, or they grudgingly decline placing it at the command of their officers. Every superintendent ought to be *au courant* with asylum literature, and he ought to be stimulated and encouraged to do something towards the advance of psychological medicine in general, or of some department thereof in particular. This institution further possesses a very complete laboratory or surgery, with all the recent introductions in the way either of drugs or apparatus—thus showing how thoroughly the medical officers keep themselves abreast of the progress of the age in regard to the medical treatment of insanity. The kitchen and laundry appeared to me to be models; I do not remember ever to have seen a finer kitchen. It is very spacious, exceedingly well lighted and ventilated, kept scrupulously clean, and furnished with the newest and most economical furnaces, boilers, kitchen-ranges, &c. The chapel is divided into neat pews, on which the Bibles and Prayer-books were laid out ready for use. The chaplain is resident, and he appears to act also as schoolmaster and to superintend the intellectual pursuits of the inmates, under the direction of the medical superintendent. I am not aware that there is any such full union of duties in the person of the chaplain in any of our British asylums. I know that in some English asylums the chaplain acts also as schoolmaster; but this, even, is found in comparatively few. Nevertheless, the combination is a good one, and one that I should like to see introduced in Britain to a fuller extent. In every asylum, it appears to me, there should be arrangements for educating such of the patients as stand in need thereof and will be benefited thereby, and for stimulating, in proper cases, and cherishing, the literary tastes of the better educated classes. My own experience teaches me that much may safely be done in this direction. The present and former chaplains of the institution with which I have the honour to be officially connected, have both offered public testimony to the advantages accruing from the introduction of the educational element in asylums.* The inmates of Gaustad, however, have neither lectures nor classes, as some of the Scotch asylums have had for years, and with such good results as to have encouraged others to follow their example. A bath-house is connected with each half of the third wing, on either side of the laundry, containing, if I remember aright, five baths, the baths being of wood. There is a plentiful and never-failing supply of water to all parts of the house, by means of an aqueduct from the Frogner-elv, a small stream rising in the hills behind, and passing near the asylum. The whole establishment is also lighted with gas, which is conveyed in pipes from the city.

Between the two halves of the front or first wing is the porter's lodge,—a handsome little edifice, which commands, as in most asylums, the principal access to the building. Between it and the "Ökonomibygning" is a spacious court, with a small circular pond and a fountain in its centre, the latter being made to play easily by turning a cock. Dr. Sandberg was kind enough to cause a display of this "water-work" in my presence and for my benefit. Certainly this is an elegant ornament at a small expense: I would gladly see ponds, stocked with gold and other fish, and ornamented with rockeries, as well as fountains, introduced in the grounds of our British asylums—especially those for the higher classes of patients—where there is an abundant supply of water from a higher elevation than the asylum or its grounds. There are still a few points, good and bad, in or about the building itself, to which I would briefly advert before leaving this section of my subject.

The medical superintendent has a separate, handsome, commodious house, healthily exposed, and commanding as fine a view, and in the same direction, as the asylum itself. This is a point too often overlooked by the managers of asylums, in their zeal for economy—I mean the provision of suitable accommodation for the physician-superintendent and his family. Too often, compulsory celibacy is enforced; he is stowed away in two or three miserable apartments in the asylum, perchance in its most noisy and bustling locality, having neither time nor place that he can call his own. This is not only cruel and unjust towards the superintendent himself, but it is a false economy and policy in regard to the management of the institution. If there is a position in life in which the most irksome and harassing routine duties should be alternated with every comfort and re-

* "Excelsior; or, Murray's Royal Asylum Literary Gazette," January, 1858—No. 4.—"Reports of our Inspectors of Schools."

laxation that life can offer or supply, it is the position of an asylum-superintendent. There is no situation, perhaps, attended with a greater degree of mental exhaustion,—none in which the sacrifice of mental and bodily vigour to the shrine of duty is more apparent, more certain. If the superintendent has not a comfortable home,—if he is immured day and night in some of the asylum cells, or, to phrase it more elegantly and pleasantly, apartments, he is little likely to remain long an efficient officer of the establishment. Indeed, he will, sooner or later, become mentally and physically unfit for the arduous duties of his office; or, if he is forced to struggle on in consequence of the *res angustæ domi*, or otherwise, he becomes a mere machine—a nonentity, void of energy, enthusiasm, and ambition—and goes his daily, weary round like a jaded horse in a mill! I do not say that such is uniformly and infallibly the case; but I do say that such is infallibly the tendency, in the circumstances I have sketched. In either view, the asylum is the loser; that is, whether there are frequent changes in the staff—for changes are not always for the better—or whether the superintendent becomes superannuated in harness. Let us hope that the managers of the new Scotch asylums will consult their own interests, as well as the best interests both of their medical officers and of their patients, by providing separate and adequate house accommodation for their medical superintendents. The female wings of Gaustad Asylum look east, while the male wings look west, the females having, therefore, decidedly the best of the view. The building, when completed, the male and female wings being symmetrical, is intended to accommodate the following number of patients:—A 18, B 50, C 35, D and E 20; or, in all, 123 of each sex—a total of 246; in round numbers, as I have already stated, 250. Due attention seems to have been paid to the provision of an ample amount of breathing space for each patient in the bed-rooms, as well as in the day-rooms. Some of the single rooms, at least, appear to contain about 2700 cubic feet of air,—a large proportion in comparison with that existing in many British asylums. There is comparatively much less space in the dormitories, however, 6160 cubic feet being allowed for a dormitory occupied by eight patients—that is, about 770 cubic feet to each. Though the practical carrying out of such an idea is necessarily expensive, I think, where it can be done from an abundance of funds, liberality and enlightenment on the part of the directors, or otherwise, that each patient should not have less sleeping room than 1000 cubic feet of air. In the pauper gallery for quiet cases, the dining saloon is so capacious as to be capable of accommodating sixty persons; and all the work-rooms, parlours, and association-rooms are comfortable, capacious, and well lighted and ventilated. The windows generally open inwards; and in their construction the architect does not appear to have been guided by the absurd idea, unfortunately too prevalent among asylum architects, that asylum windows must be guarded as strongly as those of a prison, and that every patient, if he has opportunity, will either precipitate himself suicidally therefrom, or effect his escape thereby. The result is, that the windows are much more like those of a private house than is usually the case in asylums. The accommodation provided for patients of the middle and higher ranks is particularly good. The gentlemen have their billiard-tables, and the ladies their pianos, as we have. It will be observed that the proportion of single rooms to dormitory accommodation is considerable; dormitories, indeed, are comparatively few, and are intended only for the quiet and well-behaved. This undoubtedly is one cause of the expensiveness of the asylum; but the arrangement appears to me a wise one. Dormitory accommodation diminishes expense, and a certain extent of it is undoubtedly desirable in every asylum; but it appears to me that its advantages are greatly overrated, and that, so far as the proper treatment, with a view solely to cure, of the patient is concerned, the importance of single rooms is greatly overlooked in our British asylums. The seclusion-rooms, or single rooms for the refractory and dirty, are well lighted from the top, as they ought to be. But I have great objections to the placing, in a corner of each room, a hybrid between a water-closet and night-stool, the pan of which can be removed when desired by a grating opening into the gallery. Nor do I like a gallery running along, and externally to, the top of these rooms, commanding a view from above of the actions of their inmates. This is but a clumsy and expensive substitute for the old inspection plates. Neither of these arrangements seemed to meet with the approval of Dr. Sandberg. Double bed-rooms, or dormitories for two patients, do not seem to be regarded in so objectionable a light here as they are in England. They are supposed by the English superintendents to encourage habits of sodomy and other vices; but this objection appears to me purely theoretical, at least so far as Scotland and Norway are concerned. Some of the Scotch asylums have made use of double bed-rooms for a long series of years with nothing but the best results; and so far from its being objectionable, the arrangement is extremely salutary and advisable—for instance, in the case of hysterical, timid girls, who are afraid to sleep alone, or in rooms by themselves. I should certainly strongly oppose the occupation by two persons of a bed-room properly large enough only for one—that is, containing only between 500 and 1000 cubic feet of air. But so far as my experience goes—and I am glad to find it borne out by the practice of the Norwegian

State Asylum—it is as little objectionable for two to sleep in a dormitory as for six, or any larger number, to say the least of it. The water-closets are on the self-acting principle—that is, a rush of water occurs every time the door is opened. Dr. Sandberg seemed satisfied with the arrangement, which is certainly theoretically ingenious, but has been practically found, in many British asylums, unserviceable to the extent that it is liable to get out of order, and to be made a bad use of by the patients, who sometimes take a malicious pleasure in running off all the water in the cisterns connected with such water-closets. A more extended experience may, and probably will, modify Dr. Sandberg's opinion of the usefulness of this description of water-closet, and may drive him to be content with the old and apparently clumsy system of plugs and flush-drains. The water-closets in this asylum are, moreover, much too small and inconvenient, and much too few,—both serious defects in a modern hospital for the insane. The corridors, or galleries, also, are generally too narrow, and not sufficiently cheerful, open, and well lighted. The galleries and the inspection balconies have too much of a prison appearance about them. The covered passages connecting the different wings across the airing courts are most useful in bad weather; they are suitably provided with seats. Similar arrangements are to be seen in many of our British public schools and hospitals, and are uniformly found of service.

Gaustad Asylum is chiefly intended for native Norwegians, and for curable cases; but incurable cases are not excluded if the medical superintendent states there is vacant room, and recommends their admission. Foreigners are admissible only when the form of insanity in them is curable, or when their insanity prevents their leaving the kingdom. They are admitted, however, only to the department for the middle and higher ranks, and they are charged 20 per cent. more than natives. The rates of board are regulated from time to time; but those chargeable for patients of the better ranks are more fluctuating than those for paupers, the pauper charges having been regulated by the Law of the Storthing of 17th August, 1848. From the circumstance that the institution has been but a comparatively short time in existence, fixed rates of board can scarcely be said yet to have been determined on; but they will probably, in the case of the poorer classes, at least, be pitched as low as is consistent with remuneration—profit being kept out of view as no element in the question. The rates of board include all necessaries save clothing. There are several sources of revenue to the institution, the principal being grants of money from the Storthing, the board of patients, and the profit of labour. The form necessary for admission closely resembles that followed in this country: there must be produced to the superintendent—or director, as he is called in Norway—a medical certificate of insanity, a bond for payment of board, and a requisition or application for admission. But the director has the power of waiving this ceremonial in urgent cases, at his discretion.

The asylum staff is very ample, and, apparently, very efficient, the medical details being confided to the director, assisted by a sub-physician, and several internes or clinical pupils; the director, further, having supreme control and a general superintendence over all the officers of the establishment, and over all their actings and intromissions. There is a very full and admirable code of provisional regulations (forty-eight pages 8vo),* of which the following is the Table of Contents:—Chap. I. General Regulations (*Almindelige Bestemmelser*). Chap. II. House Regulations (*Husorden*). Chap. III. Special instructions: for A. Director or Physician-Superintendent,—an officer who holds the same position as the medical superintendent, or resident medical officer, of the British asylums:—B. Assistant Medical Officer (*Reservelægen*):—C. Internes, or clinical assistants or clerks (*Candidaterne*):—D. House Steward (*Forvalteren*):—E. Treasurer or Cashier (*Kassereren*):—F. Matron and Head Attendant (*Matronen og Overvorgteren*):—G. Attendants (*Vogterne*):—H. Head Laundress (*Oldfruen*):—I. Housekeeper (*Husholdersken*):—K. Engineer (*Maskinisten*):—L. Barber (*Barberen*):—M. Farm Overseer (*Avlskarlen*):—N. Gardener (*Gartneren*):—O. Porter (*Portneren*):—P. Night Watchers (*Nattevogterne*):—Q. Cooks (*Kjökkenpigerne*):—R. Laundresses (*Vaskepigerne*):—S. Messengers (*Budeierne*):—T. Stoker (*Fyrböderen*):—U. House Servant (*Budet-Huskarlen*):—V. Farm Servant (*Gaardskarlen*). The foregoing enumeration is sufficient to indicate that the staff is in some respects, if not in all, a model one; and in regard to its completeness, it is certainly worthy of imitation in this country. There may be an approximation to such a staff in some of our larger asylums; but I am not aware of any asylum of similar size—accommodating, at present, only between 160 and 170 patients—with anything like so perfect a machinery for its due management. Such a machinery, however, cannot be maintained unless at a great expense; and this must be borne in mind in questions affecting the cost of erecting and maintaining Gaustad Asylum.

The salary of the superintendent is, I believe, about 1600 to 1800 specie dollars, or about 400*l*. a-year English, with a handsome house, as I have already mentioned. This allowance is certainly not too much: perhaps it may be increased when the establishment possesses its complement of patients. He has the

* "Midlertidigt Reglement for Gaustad Sindssygeasyl. Approberet ved Kongelig Resolution af 29de August, 1858."

control—as every superintendent ought undoubtedly to have—both of the medical and fiscal departments of the management. All the officials are under his orders; and he has the privilege and power of appointing all save the chaplain, medical assistant, steward, and treasurer, whom he merely nominates. It will thus be noticed that the matron is not placed on the same footing as in this country; she occupies altogether a subordinate position. The principle of having her appointed by, and entirely subordinate to, the medical superintendent, is certainly the right one. This arrangement avoids the collision of conflicting powers and positions which not unfrequently occurs under other circumstances. The superintendent or his substitutes give all necessary information regarding the admission, &c., of patients, and grant all requisite certificates; he conducts all correspondence; keeps certain registers, or "protocols," as they are called in Norway, and draws up certain reports and statistics, conformably to the law of 17th of August, 1848, sect. 5 and 6. He gives clinical instruction to his internes, or pupils—an excellent arrangement, worthy of all imitation. Thanks to the regulations for admission into the medical service of the East India Company, several of our public asylums now give courses of clinical lectures on insanity and its treatment—for example, Bethlehem and St. Luke's in London, the Royal Asylums of Edinburgh and Aberdeen, and the District Asylum at Belfast; but otherwise there is little or no arrangement for clinical teaching. The opportunities of hospitals for the insane are not duly made available, and the consequence is, that comparatively few medical practitioners know anything practically of the proper treatment of insanity, and of the construction and management of hospitals for the insane. Every asylum, in my opinion, ought to have, in proportion to its size, one or more clinical assistants, who may be resident or not, according to circumstances, but some of whom, I think, should be resident, and others not, just like the internes and externes of the Parisian hospitals—the resident and non-resident clerks of our British general hospitals. They might be appointed for limited periods, so as to ensure a succession of fresh, energetic, and enthusiastic pupils and assistants. Such an arrangement would possess many advantages. Every asylum would become a psychological school, and would send forth medical practitioners conversant with a department of medicine of which they are at present deplorably ignorant—psychological medicine; the pupils would act as assistants to the superintendent, who would be thus relieved of a vast amount of drudgery in the writing out of cases, drawing up of statistics, compounding of drugs, and in minor surgical operations, &c.; while the presence of pupils, and the existence of other similar hospital-schools, would stimulate him to keep himself *au courant* with the progress of psychological and medical science, and to use his best efforts to maintain the reputation of his particular asylum or school, or to outdistance his compeers in a friendly competition. The arrangement would not be a costly one; no difficulty would be experienced in getting candidates for these appointments; students of medicine would eagerly offer their services in exchange for the opportunities offered, especially in asylums of superior reputation, or in the larger or university towns. At least once a month the superintendent of Gaustad Asylum calls together a council, consisting of the chaplain, assistant-physician, steward, and treasurer, and such of the subordinate officers as he sees fit, for the purposes of conference and instruction. This must afford him a good opportunity of explaining his views, which views are much more likely to be carried fully into effect if they are intelligently understood than otherwise. His other duties and powers are so similar to those of asylum-superintendents in this country, that they need not here be enumerated.

The assistant-physician is appointed for three years; he is removable on three months' notice, and he cannot leave without giving three months' intimation. He is invested with plenipotentiary powers in the absence of the superintendent. He receives and examines new cases, determining to what part of the house they are to be sent, &c.,—subject, however, to the approval of the superintendent. He specially supervises all subordinate officers; performs *post mortems* and simple surgical operations; keeps certain books; and assists the superintendent, if required, in correspondence, the drawing up of reports and returns, in chemical investigations, and otherwise. The surgery is under his charge, with the drugs, re-agents, apparatus, and surgical instruments. He acts as librarian, is conservator of the scientific and literary library, and draws up the catalogue. He sees specially that the internes, matron, and head-attendant discharge their respective duties.

Three "internes," or clinical pupils, are appointed by the superintendent as assistant medical officers. They hold office for one year; and the appointments are so arranged, that only one falls to leave or resign his office at a time. They are subject to the superintendent and assistant-physician; they accompany them in their daily visits to the galleries,—just as the clinical clerk follows his physician or surgeon in walking the wards of a general hospital,—carry out their medical instructions, keep journals of the cases, write prescriptions to dictation, do the bandaging, assist in minor surgical operations, and superintend and instruct the attendants in bandaging, the application of blisters, and similar operations. They likewise assist in making out registers of patients, lists of medicines, statistics of disease, &c. They have exercises in psychological medicine prescribed to them by the director or his assistant. After the daily medical visit, one of the internes is placed in charge for the day; and it becomes his duty to look after the giving out of medicines and surgical appliances, to order supplies of medicine from town, and to see that the medicines are properly distributed, and the prescriptions or directions of the superintendent and assistant-physician duly carried into effect. In urgent cases, in the absence of the superior medical officers, the interne in charge for the day acts as their deputy, and does what he considers necessary. He is precluded leaving the institution during his day of charge, unless either the assistant-physician or another interne take his place. This secures that a medical officer is always to be found in the institution—another admirable arrangement, worthy of being followed in this country, and an additional advantage springing from the appointment of clinical assistants. An interne may obtain leave of absence for a day from the assistant-physician; but, if he desire a longer holiday allowance, he must apply to the superintendent.

The house-steward is an officer of considerable importance; and his appointment, as well as that of a resident treasurer, relieves the superintendent of an immense amount of most irksome mechanical drudgery, which would otherwise, in great measure, dissipate time that is more worthily and profitably employed. There is no doubt that the work laid upon the shoulders of many of our asylum superintendents in this country should properly be distributed among three officers—superintendent, treasurer, and clerk. His talents and skill should not be misapplied, his energies unprofitably dissipated, and the natural order of things reversed, as at present. The steward has immediately under his jurisdiction the matron, head-attendant, laundresses, housekeeper, attendants, farm overseer, engineer, gardener, porter, night-watch, and other servants of whatever kind.

The treasurer, like the steward, is appointed by the Department for the Interior (*Departement et for det Indre*), on nomination by the superintendent, and he is bound to give 1500 specie dollars security. Besides fulfilling the proper duties of a treasurer, he acts as bookkeeper and secretary, and checks, to a certain extent, the steward. He has an office in the institution, the business hours being 9 A.M. till 2 P.M. He does not necessarily reside in the building, but is allowed, if he prefer, to live somewhere in the vicinity.

The machinist or engineer has charge of the apparatus for water and gas supply, for heating, and also of the sewers; and he does any smith-work required about the house. When the asylum possesses a smith's forge, as it contemplates doing, this will also be placed under his jurisdiction. He is directly under the control of the steward, as are also the gardener and farm overseer. There are both a male and female night-watch; but I am not aware how far this arrangement has been found to answer. The proportion of attendants to patients is as 1 to 6 or 8. This is a large and favourable proportion in comparison with that to be found in some of our older asylums, especially, where the proportion falls so low as 1 in 10, 15, or even 20. This large proportion, of course, adds materially to the cost of maintaining the establishment, and is another item of the expense of construction and management to which I have already more than once alluded.

Let us now return to Dr. Sandberg's First Annual Report, and cull therefrom some particulars regarding the statistics of the institution, and the treatment—dietetic, medical, and moral—of its inmates. The statistical tables, with which the Report is abundantly furnished, are based on a classification or division of the inmates into pauper patients, or those paid for by parishes or districts, and private patients, or those paid for by private friends. This division is, to a certain extent, a useful one; it is now being introduced into the statistical reports or tables of the Scotch asylums. The total number of patients under treatment during the year 1856 was 219—132 belonging to the pauper class, 76 males and 56 females; and 87 to the private class, 50 males and 37 females. The following were the native districts of these patients:—

| | | | |
|---|---|---|---|
| Akershus Amt | 44 | Grimstad | 1 |
| Jarlsbergs Amt | 25 | Lister and Mandals Amt | 4 |
| Christiania | 18 | Sarpsborg | 3 |
| Bratsberg Amt | 18 | Skien | 1 |
| Hedemarkens Amt | 22 | Kongsberg | 2 |
| Nedenæs Amt | 12 | Drammen | 6 |
| Smaalenenes Amt | 10 | Bergen | 2 |
| Budskeruds Amt | 12 | Nordre Bergenhus Amt | 2 |
| Christians Amt | 9 | Nordlands Amt | 1 |
| Frederikshald | 3 | Soon | 1 |
| Laurvig | 3 | Kragerö | 2 |
| Aalesund | 1 | Svelvig | 1 |
| Arendal | 1 | Brevig | 1 |
| Christiansand | 1 | Flekkefjord | 3 |
| Röraas | 1 | Christiansund | 1 |
| Romsdals Amt | 2 | Kobbervig | 1 |
| Throndhjem | 2 | Stavanger | 1 |
| Frederiksstad | 2 | | |

The ages of those under treatment were:—

| | | | |
|---|---|---|---|
| Under 20 years | 11 | Between 50 and 60 | 31 |
| Between 20 and 30 | 71 | „ 60 and 70 | 4 |
| „ 30 and 40 | 64 | „ 70 and 80 | 2 |
| „ 40 and 50 | 36 | | |

The duration of the disease, or of the last attack thereof, prior to admission, was:—

| | M. | F. | | M. | F. |
|---|---|---|---|---|---|
| Under ½ year | 31 | 29 | From 5 to 6 years | 4 | 2 |
| From ½ to 1 year | 22 | 19 | „ 6 „ 7 „ | 2 | 1 |
| „ 1 „ 1½ „ | 11 | 9 | „ 7 „ 8 „ | 4 | 3 |
| „ 1½ „ 2 „ | 7 | 2 | „ 8 „ 9 „ | 4 | 2 |
| „ 2 „ 3 „ | 18 | 4 | „ 9 „ 10 „ | 3 | 1 |
| „ 3 „ 4 „ | 9 | 8 | „ 10 „ 15 „ | 3 | 5 |
| „ 4 „ 5 „ | 3 | 6 | „ 15 „ 20 „ | 3 | 2 |

In regard to relapses, or multiple attacks, 14 males and 16 females had had 1 previous attack; 8 males and 2 females had had 2; 2 males and 1 female, 4; and 5 males and 7 females, several.

The causes of insanity in the 219 patients under treatment were as follows:—

| | M. | F. | | M. | F. |
|---|---|---|---|---|---|
| Hereditary | 17 | 16 | Typhus | 1 | 3 |
| Onanism | 26 | 4 | Imprisonment | 3 | 0 |
| Disappointments in love | 13 | 10 | Disorders of digestive system (fordöielsesbesvær) | 2 | 1 |
| Intemperance and intoxication | 19 | 3 | Apoplexy | 2 | 0 |
| Domestic sorrows | 5 | 17 | Blood-letting | 2 | 0 |
| Religious scruples | 9 | 5 | Remorse of conscience | 2 | 2 |
| Vexation (ærgrelse) | 11 | 2 | Uterine diseases | 0 | 2 |
| Fright | 3 | 7 | Home-sickness | 0 | 2 |
| Syphilis, and mercurialization | 10 | 1 | Illicit intercourse (besvangring) | 0 | 2 |
| Over-exertion (overanstrængelse) | 9 | 0 | Disease of brain | 0 | 2 |
| Pregnancy and parturition | 0 | 9 | Phthisis (tuberkulose) | 0 | 2 |
| Destitution and its cares (næringssorger) | 3 | 3 | Jealousy | 1 | 0 |
| Bodily ill-usage | 2 | 2 | Measles | 1 | 0 |
| Lesion of head | 3 | 1 | Bronchitis | 0 | 1 |
| | | | Pneumonia | 0 | 1 |

Dr. Sandberg divides causes into psychical and physical; but he apparently finds the same difficulty that we in Britain do in discovering special causes, or causes at all, or in determining that, in any given case, the cause belongs purely to the one category or to the other. Hereditary transmissibility is well illustrated by the next Table, which shows in what relatives insanity had previously developed itself:—

| | M. | F. |
|---|---|---|
| Great-grandfather (oldefader)* | 1 | 0 |
| Great-grandmother (oldemoder) | 2 | 0 |

* As showing the composition and etymology of Norwegian words, and their general resemblance to the English—or rather the Scotch—I have given in the above Table the Norsk words for the persons or relational terms therein expressed. Those who are etymologically inclined, or who are fond of the philosophy of language, will find some most interesting studies in the Scandinavian languages; for instance, in the Icelandic, or original Norsk, in the Norwegian of to-day, which is a modification of the Danish, and in the Danish itself. I had the pleasure of a most interesting discussion on the analogies of the Norwegian and Scotch languages

| | M. | | F. |
|---|---|---|---|
| Paternal grandfather (farfader) | 4 | ... | 1 |
| Paternal grandmother (farmoder) | 2 | ... | 1 |
| Maternal grandmother (mormoder) | 4 | ... | 4 |
| Father (fader) | 9 | ... | 2 |
| Mother (moder) | 13 | ... | 12 |
| One or more brothers (brödre) | 7 | ... | 12 |
| One or more sisters (söstre) | 4 | ... | 5 |
| Paternal uncle (farbroder) | 4 | ... | 2 |
| Maternal uncle (morbroder) | 4 | ... | 4 |
| Paternal aunt (faster) | 6 | ... | 3 |
| Maternal aunt (moster) | 5 | ... | 4 |
| More distant relatives | 11 | ... | 10 |

The influence of the mother in transmitting the predisposition to insanity to her offspring is here abundantly evident.

The admissions during the year appear to have amounted to 157—92 males and 65 females. In regard to admissions, the superintendent seems to have much more real power than with us. No sheriff, justice of the peace, clergyman, or elder stands between him and the applicant for admission. He has certainly to do with a "Control Commission," of which more anon. The method of procedure, I conceive, approaches much nearer simplicity, and consequently efficaciousness, than either the present or the past Scotch system. The form of insanity in the patients admitted was:—

| | M. | | F. | | Total. |
|---|---|---|---|---|---|
| Melancholia | 37 | ... | 34 | ...... | 71 |
| Mania | 26 | ... | 17 | ...... | 43 |
| Stupidité | 4 | ... | 0 | ...... | 4 |
| Delirium tremens | 2 | ... | 0 | ...... | 2 |
| Dementia | 17 | ... | 13 | ...... | 30 |
| Idiotism (acquired) | 1 | ... | 0 | ...... | 1 |
| Epilepsy with mania | 0 | ... | 1 | ...... | 1 |
| General paralysis | 3 | ... | 0 | ...... | 3 |

The prevalent form of insanity appears, therefore, to be melancholia; the sexes are nearly equally affected, the males slightly predominating. It constitutes nearly one-half of the whole cases admitted, and is more than equal to both mania and dementia conjoined. So far as I could learn, melancholia seems to be also the most common form of insanity throughout Norway. This has been attributed—with what justice I know not—to the facts that the population is thinly scattered, that the people live at great distances apart—isolated, as it were—greatly shut out from intercourse with the world, surrounded by gloomy mountains, and by what was called by a young Norwegian, whom I once with two of the most distinguished linguists and philologists in Norway—the celebrated Professor P. A. Munch, author of "Det Norske Folks Historie," and Professor C. R. Unger, both of the University of Christiania, the former Professor of History, the latter of Modern Languages.

encountered on the summit of Ben Nevis, "wild nature." Mania ranks next in order of importance. The sexes are affected unequally—26 males to 17 females, nearly double. Dementia follows. Here, again, the sexes are nearly equally affected; the males, however, again predominating. But, in estimating the relative frequency with which the sexes were affected, it is necessary to bear in mind that a much larger proportion of men was admitted than women—92 to 65. The numbers given above relate to the absolute admissions, not to their comparative frequency as to sex. General paralysis occurs in 3 cases, all males. This is in conformity with our experience in this country. Delirium tremens occurs in 2 males. It does not appear whether these cases were admitted by mistake, or whether delirium tremens is regarded in Norway as a form of insanity. I see no reason why, to a certain extent, it should not be so considered, and rendered liable to all the disabilities of insanity. Medical jurists will find a difficulty in establishing any valid difference between delirium tremens and insanity, unless that, in the former case, the paroxysm or attack is generally of much shorter duration. In classifying the forms of insanity, Dr. Sandberg recognises primary and secondary forms. The former are marked either by mental depression or exaltation, and they may be either simple or complicated. His typical or primary forms are melancholia and mania. A very common complication of the primary forms is stupor: hence we have melancholia with stupor, or stupidité—a well-marked form of insanity. The secondary forms are characterized either by confusion of ideas (*forvirring*) or by dulness of intellect (*slövhed*). They include dementia (the "*wahnsinn*," "*verrücktheit*," and "*verwirrheit*" of the German psychologists) and idiocy, with their complications, among which are epilepsy and general paralysis. In regard to the proportion in which the two great classes of patients—pauper and private—were affected with the chief forms of insanity—

| | Pauper cases. | Private cases. |
|---|---|---|
| Melancholia occurred in . . . . . . . . | 30 | 41 |
| Mania " . . . . . . . . | 29 | 14 |
| Dementia " . . . . . . . . | 21 | 9 |

The discharges during the year amount to 65—less than one-half the admissions. But it was to be expected that the latter should largely preponderate over the former in a new institution. The cured, relieved, and improved stood in the following proportions:—

| | Pauper. | Private. | Total. |
|---|---|---|---|
| Cured . . . . . . . . . . . | 16 | 13 | 29 |
| Improved . . . . . . . . . . | 1 | 6 | 7 |
| Uncured . . . . . . . . . . | 12 | 17 | 29 |
| Total . . . . . . . | | | 65 |

The recoveries are thus 29, in relation to 65 discharges, 157 admissions, or 219 patients under treatment during the year. Of 29 recoveries, 18 were females and 11 males. The recoveries occurred at the following ages:—

| | M. | | F. |
|---|---|---|---|
| Under 20 years | 4 | ... | 0 |
| Between 20 and 30 | 5 | ... | 2 |
| „ 30 „ 40 | 1 | ... | 6 |
| „ 40 „ 50 | 3 | ... | 0 |
| „ 50 „ 60 | 2 | ... | 2 |

The duration of insanity in the same cases was:—

| | M. | | F. |
|---|---|---|---|
| Under ½ year | 6 | ... | 6 |
| Between ½ and 1 | 5 | ... | 3 |
| „ 1 „ 2 | 2 | ... | 1 |
| Three years or upwards | 1 | ... | 0 |

In connexion with the subject of discharges, it is necessary here to mention that no patient is allowed to remain in the asylum longer than two years, as a general rule; but exceptions may be made by the superintendent, if the interests either of the patient or of the institution seem to require them. The deaths were 6, 5 males and 1 female, or 4 pauper and 2 private cases—a sufficiently small mortality.*

One of the most important subjects in connexion with the treatment of the patients in this asylum is that of the dietary regulations. I append hereto a table and accompanying explanations, not merely for the purpose of showing that the patients, and especially the pauper patients, are very well cared for as to food, but also with a view to indicate the nature of the Norwegian articles of diet as contrasted with our own. Some of these are peculiar to Norway, or common to it with other countries of Northern Europe, but little known in England, though one of them at least is comparatively familiar in Scotland. The one in question is the national dish, "Gröd," which is equivalent to the Scotch porridge; it forms the bulk of the food of the peasantry, and a remarkably good food it is. The native Norwegians generally use water-gröd, made of oat or barley-meal and water, and supped with milk; but for strangers, if they consent to make it at all—for they regard it as unfit for the fastidious palate of the English tourist—they make it with milk or cream, flavouring it with carraway, and doing it up with oil, &c. I preferred infinitely the plain water-gröd, and I found it frequently a most satisfactory meal—where, it must be confessed, nothing else was sometimes to be had—after a day's journey on foot or horseback on the bleak

* Full tables of the admissions, discharges, deaths, &c., will be found in one of the schedules appended to Dr. Sandberg's Report, entitled, "Extract af Gaustad Sindssygeasyls Personalprotocol for Aaret 1856."

Norwegian Fjelds. Rye bread is another national article of diet—the staple home-baked bread of the poor Norwegian. In the towns, white or wheaten bread may be had, though it is greatly dearer; but in the interior, nothing in the shape of bread can be had except rye bread and various forms of cakes, resembling Scotch oatmeal cakes, made of oat or barley-meal, variously seasoned. The rye bread has, to the stranger as yet unacquainted with it, a most forbidding or repulsive aspect; it is very dark, like the black bread of the Spartans—heavy, moist, and sour. The natives prefer it sour and moist, and make it so intentionally; and they bake very large quantities at a time—sufficient to last over weeks or months, as the case may be. It varies greatly in character in different parts of the country and in different families, some making it sweeter, lighter, and drier than others, according to taste. Many of the sport-loving Britons who, every summer, frequent the Norwegian Elvs to fish the salmon, or the Norwegian Fjelds to shoot the reindeer or bear, get rye bread baked specially for themselves, sufficiently sweet to be palatable. I had some difficulty in resigning wheaten bread on leaving Christiania for the interior; but, after sundry disagreeable gastric symptoms, I soon became fond of it, or rather the stomach exhibited a tolerance of it for the days and weeks during which it became necessary to subsist mainly on this fare. Previous acquaintance with it in Germany rendered this less difficult than might otherwise have been the case. In the latter country I have seen it so coarse that horses and men fed equally upon it. Yet a third dietetic peculiarity of the Norwegians is their cheese, particularly their "ged-ost," or goat's-milk cheese—for they are great cheese-makers and cheese-eaters, and have an infinity of kinds. This is very sweet, and, to my taste, nauseous; in Norway, however, it is evidently regarded as a great delicacy. But, to return to the dietary of the Gaustad Asylum, I am bound to confess that it appears to me remarkably good. I speak particularly of the pauper, or lowest, scale of dietary. That for the higher classes is as ample and varied as it could be in a private home; while that for the paupers is infinitely better. For if we contrast the asylum dietary with the ordinary food of the poorest classes of Norwegians, especially those in remote country districts, living far from supplies from towns, and subsisting almost entirely throughout the year on rye bread, gröd, and milk, with cheese and butter, seldom tasting animal food, there is great reason to commend the liberality of the managers of Gaustad. These gentlemen evidently regard insanity as a disease dependent on, or connected with, impaired nutrition, and requiring, therefore, for its proper treatment, as a basis for all medical or moral means of cure, a diet both good in quality and full in quantity. Every day the patients have coffee or tea, bread and butter, for breakfast: the proportions being $\frac{3}{4}$ lod—the Danish "Lod" being equal to our $\frac{1}{2}$ oz.—coffee-beans, or $\frac{1}{6}$ lod tea, $\frac{3}{4}$ lod Havannah sugar, and $\frac{1}{2}$ pægel—a pægel being equal to about $\frac{1}{2}$ pint English—of milk, 16 lod rye bread, and $1\frac{1}{2}$ lod butter. The higher classes have also a portion of white or wheaten bread (*hvedebröd*). At dinner, again, there is considerable variety of meat and fish, fresh and salt, with soups and vegetables of different kinds. The dietary is certainly both more ample and more varied than that of most of our district or pauper asylums in this country. The error, if error it be, in our asylums is rather in the form of too great sameness than of too small allowance; but the one is sometimes as productive of serious effects as the other evil. I know not what dietary regulations may be established in the new Scotch asylums; but it is to be hoped the General Board of Lunacy for Scotland will not be content with a minimum standard. The patients in Gaustad Asylum have three regular meals—breakfast, dinner, and supper; but all who work, or otherwise deserve or require them, have an "*eftermiddagsmad*," or afternoon lunch, between dinner and supper, and a "*mellemmiddagsmad*," or forenoon lunch, between breakfast and dinner; and as the majority of the patients work, there are virtually four meals daily in winter, and five in summer. The hours for meals are as follows:—

| | | |
|---|---|---|
| Breakfast . . . | 6 A.M. | in summer. |
| „ . . . | $7\frac{1}{2}$ „ | in winter. |
| Dinner . . . | 12 noon | for pauper patients. |
| „ . . . | 1 P.M. | for private or higher class patients. |
| Supper . . . | 7 „ | in winter. |
| „ . . . | 8 „ | in summer. |
| Eftermiddagsmad . | 4 „ | for workers. |

The record of the industrial department (*arbeidvirksomhed*) is very interesting and satisfactory, and shows the authorities of the Christiania State Asylum to be fully alive to the importance of occupation—of useful and productive labour—both as a means of cure and as a source of profit. But the Norwegians go farther than we do in regard to the prescribing of occupation or labour for their patients. Work with them is rendered compulsory (I presume the regulation refers chiefly to the pauper classes), and the indolent and refractory are punished—even with restraint or seclusion, as it would appear. This restraint or seclusion is recommended to be as short and as mild as possible; but, query, to what extent is it at all necessary or judicious? Most authorities agree as to the advisability, or at least harmlessness, of holding out rewards to the insane for good conduct and industry; but the subject of punishment is one, to say the least of it, which is usually avoided as a difficult and delicate one. I do not intend here to open up the question; but I am bound to

*Table showing the Dietary Regulations of the State Asylum of Norway at Christiania.**

| Days of the Week. | Dietary for better Classes. (Bedre Forpleining.) | Ordinary Diet for Pauper Classes. (Simpel Forpleining.) |
|---|---|---|
| | 1. *Morning* (*Breakfast*). | 1. *Morning* (*Breakfast*). |
| Daily . . . . | *Patients* have tea, bread and butter, with cheese, or some substitute. *Attendants* have coffee, bread and butter, with cheese, or some substitute. | *Patients* have tea, butter, and bread. *Attendants* have coffee, butter, and bread. |
| | 2. *Mid-day* (*Dinner*). | 2. *Mid-day* (*Dinner*). |
| Sunday . . . . | Roast meat and pudding. | Fresh meat and meat soup. |
| Monday . . . | Sago soup and stockfish (klipfisk). | Milk broth (melkevelling, or melk-suppe) and stockfish. |
| Tuesday . . . | Fresh meat with horseradish sauce, and meat soup, with rice. | Fresh meat and meat soup. |
| Wednesday . . | Fresh fish and rice pudding (risvelling). | Fresh fish and fish soup. |
| Thursday . . . | Peas, salt meat, and bacon, with stewed greens or potatoes. | Peas, salt meat, and pork. |
| Friday . . . . | Beef-steak and sago-pudding (sago-velling). | "Menagesuppe" and "Lapskous." |
| Saturday . . . | Beer-soup (öllebröd), herring, and pancakes. | Beer-soup and herring. |
| | 3. *Evening* (*Supper*). | 3. *Evening* (*Supper*). |
| Tuesday & Friday | Porridge (gröd) and milk. | *Sunday and Thursday*, tea, butter, and bread. |
| Other days . . | Tea, bread and butter, with meat, &c. | Porridge (gröd) and milk. |
| | *Afternoon Lunch*† (*Efter-middag*). | *Afternoon Lunch*† (*Efter-middag*.) |
| Daily . . . . | Half portion of butter, bread, and coffee. | Half portion of butter, bread, and coffee. |
| | *Forenoon Lunch*† (*Mellem-middag*). | *Forenoon Lunch*† (*Mellem-middag*). |
| Daily . . . . | Half portion of butter and bread. | Half portion of butter and bread. |

* In connexion with, and in explanation of, this table, it will be necessary to give some particulars regarding the Norwegian or Danish dishes therein named. Comparatively full details as to the composition of these dishes, and of the proportions of materials used, will be found in one of the tables appended to Dr. Sandberg's Report: "Uddrag af det fortiden gjældende Spise-reglement for Gaustad Sindssygeasyl."

The weights of solids are given per "Lod," a Danish weight equal to our ½ oz.

The liquid measure generally used is the "Pot," a Danish measure equal to somewhat more than our quart.

"Gröd" is a national dish, equivalent to the Scotch "porridge," or "stirabout." It is generally made either of oat or barleymeal, with water or milk; but it is sometimes also eaten with oil, spices, cream, &c.

About 6 oz. or 12 "lod" of fresh meat, freed of sinews and bones, are allowed per patient.

Meat soup contains, per patient, 1 oz. meat, groats, greens, &c.: "Lapskous" contains 4 oz. of meat: "Menagesuppe" contains groats, syrup, gravy, &c.: Fish soup (fiske-suppe) contains barleymeal, milk, greens, &c.: Beer-soup (öllebrôd) is made of beer, milk, barleymeal, and syrup, according to Dr. Sandberg's Report, but of beer and grated rye-bread according to the Danish Dictionary of Ferrall and Repp. Gryngröd, or Gryns-gröd, according to the Dictionary, is simply boiled groats, milk or beer, and sugar. "Byggryn" is barley-groats: "Byg-suppe" is barley-broth: "Klipfish" is common saltfish, or stockfish.

The private patients of the higher ranks have potatoes at certain times in addition to, or in lieu of, other vegetables; but the potato appears to be a comparatively scarce vegetable in Norway. They have also refined sugar to some of their dishes, *e.g.*, to coffee, instead of raw or coarse sugar, and cream instead of milk.

Their "Smorrebröd," or butter and bread, consists of 12 lods of rye bread, or 6 of wheaten bread, or 8 lods of former and 4 of latter, with 2 lods of butter. On Sundays, festival days, and some other occasions, there is an addition of cheese with other extras.

Their rice pudding (riisbudding) contains rice, milk, eggs, coarse sugar, raisins, butter, potato-meal, lemon-peel, &c. The "Sago-suppe" consists of sago, cherries, raisins, coarse sugar, &c. Pancakes are made of wheat flour, butter, milk, eggs, and refined sugar; sauce for roast meat, of butter and wheat flour; and horseradish sauce, of horseradish, wheat flour, butter, coarse sugar, &c.

† Are supernumerary meals, only given to the workers and such other patients as the superintendent prescribes them for.

express my belief that, though the printed regulations of Gaustad Asylum contain clauses to the above effect, they are not, to the full extent, acted upon; for I presume, in regard to the imposition of restraint or seclusion, Dr. Sandberg possesses and exercises a humane and wise discretion. I quote from the Regulations of the Asylum the clauses to which I refer, as the testimony of Norwegian psychologists on the subject of compulsory work, and the punishment of the indolent and refractory:—

"Enhver Syg maa beredvilligen deltage i de Arbeider, som Asylet til hans eget Bedste fordrer af ham, være sig paa Marken, i Haverne, i Anlæggeneeller hvor det maatte ansees hensigtsmæssigt."

"Uvillige og gjnstridige Syge ville paalægge Bestyrelsen den tunge Pliget at anvende Tvangströie, Indespærren i Eneværelse," &c.

"Men disse Tvangsmidlers Anvendelse skal være saa kortvarig og lempelig som muligt."*

But, though compelled to work, the patients are not compelled to work for nothing. The superintendent possesses the power, at his discretion, of giving rewards to the industrious, either in the form of small necessaries or gifts, or as sums of money when the patient leaves the institution. The placing to the credit of patients of the value of their labour; the laying up of their earnings during their residence, as it were, in a savings'-bank; and the handing over to them, directly or indirectly, on their leaving, of the accumulated sum, or the balance thereof—for it is but fair that the patient should defray, in whole or in part, the cost of his maintenance and treatment—seem to me to constitute an admirable arrangement. The poor artisan or labourer is thus provided with a sum sufficient to start him afresh and satisfactorily in the world, or to keep him comfortably until he succeed in obtaining suitable employment. This principle is acknowledged in the Reserve Funds of some English asylums, from which grants of money are made to deserving cases on their leaving the asylum to resume the struggle of life. But it is a principle which should be uniformly acted upon; if it is good in one asylum, it is, or would be, equally serviceable in another. It is, indeed, a new development of the principle of life insurance, and a development to which I would urgently direct the attention of the authorities of our lunatic asylums. Such a provision would lessen the pangs of confinement to many an industrious tradesman, who would experience great satisfaction in the assurance that he was supporting himself, or largely contributing to his own support—a burden neither upon friends nor parish. Removal from an asylum is frequently a curse instead of a blessing; it is a step from comparative affluence, ease, and comfort, to misery and beggary; and every superintendent of an asylum can readily recall to memory

* "Midlertidigt Reglement for Gaustad Sindssyge-asyl," p. 5, sect. vii.

illustrations and cases corroborative of this statement. I know no more worthy object of the charity of the rich than such reserve funds attached to our large public asylums. It may appear hard and unjust to compel patients to work; but, that compulsory work—where, of course, voluntary work cannot be substituted—in the case of pauper patients is generally a direct benefit to patient and asylum alike, I have no hesitation in asserting. I had occasion to try the experiment lately. Four powerful men in the prime of life—all cases either of acute or paroxysmal mania, and all both destructive and violent—had for a considerable period caused much trouble and expense by breaking windows, tearing up clothes, and destroying the walks and walls of the airing courts. An attendant was engaged specially to superintend these men, who had a court allocated to themselves for exercise. Still the violence and destructiveness continued; no kind of dresses was found strong enough to resist their mischievous propensities; one man tore up, with apparent ease, the stoutest canvas and moleskin, as well as locked boots. I now resolved to direct their physical force into some useful channel, and I accordingly set the four men, under charge of their attendant, to pump-work. They were engaged in this hard, but regular, mechanical employment from 9 A.M. till ½ past 12; and again from 2 to 4 or 5 P.M. daily. The result was almost immediate and satisfactory, consisting in comparative quietude, good behaviour, improved sleep and appetite. The experiment continues to work well after an interval of several months. In such cases, independently of the good done to the patients, I doubt not that the asylum would ultimately be no loser, inasmuch as against the wages and keep of an attendant are to be placed, as per contras, the enormous amount and considerable value of clothes destroyed and windows broken. The kind of work engaged in by the patients of Gaustad appears to be chiefly the following:—Cutting firewood, felling trees, gathering or teasing hair, household work, field and agricultural work, shoemaking, carpentry, mason work, bookkeeping, wool work,—gathering, carding, and spinning of wool, stocking-knitting, and needlework. While the men are chiefly employed in out-door labour, field and garden work, cutting timber and splitting firewood, or in in-door handicrafts, the females are engaged in cooking, household work, wool work, needlework—in making or mending stockings, shirts, jackets, trowsers, coats, petticoats, night-dresses, under-clothing, handkerchiefs, window-blinds, &c. More women seem engaged in wool work than in any other single department of female handicraft—upwards of ten daily—while only four are occupied in household work, and three in sewing. The quantity of work done by the females may be estimated by the following list:—

| | | |
|---|---|---|
| 15 Pairs men's boots. | 127 Men's shirts. | 30 Coats. |
| 74 Pairs women's boots. | 60 Chemises. | 84 Night-caps. |
| 66 Pairs men's shoes. | 48 Pairs trousers. | 18 Night-dresses. |
| 12 Pairs women's shoes. | 20 Jackets. | 109 Window-blinds. |
| 207 Pairs stockings. | 39 Petticoats. | 121 Handkerchiefs. |

The women would thus appear to do a considerable amount of tailoring. Full statistical tables are given in Dr. Sandberg's Report, the accuracy of which is vouched for by a "Control Commission," consisting of three persons, who act as auditors of accounts. The males are chiefly employed felling timber and cutting it up into battens, or firewood: a large amount of birch, pine, and other woods has thus been hewn and cut up during the year. This is no more than we should expect in a timber-producing country like Norway. Agricultural or garden work stands next in order of importance; the spring and harvest seasons appear to be by far the busiest to this section of the workers. I saw many patients engaged in reclaiming waste lands and laying out airing courts, for the grounds belonging to the asylum are as yet in a comparatively wild and uncultivated state. Shoemaking is an important handicraft: in this department the patients have the benefit of the supervision of a shoemaker and his apprentices. Carpentry is a sister trade of equal importance; all the garden and agricultural implements are made and repaired in the carpenter's shop, and all the ordinary house jobbing done by the wrights or joiners. A few of the males were engaged in special occupations: one man, during my visit, was painting the walls of some of the parlours and corridors most tastefully. In connexion with the hours of labour, I may here mention that the patients go to bed at half-past nine P.M. in summer, and at half-past eight in winter—rather later, in both cases, than in most British asylums, where a common source of complaint is the hardship of being compelled to go to bed in summer by daylight (*e.g.*, at eight P.M.).

I must now approach, with all due caution and respect, a subject which is in great measure tabooed or passed over in solemn silence in most of our British Asylum Reports—I mean the subject of restraint and seclusion. In a table appended to Dr. Sandberg's Report, we find a description of the nature of the restraint and seclusion employed in the Christiania State Asylum, their duration, and the necessity or causes for their employment.*

* "Extract af Gaustad Sindssyge-asyls Behandlingsprotokol for Aaret 1856:"

1. Symptoms, or conditions, on account of which restraint was rendered necessary (*sygdoms-symptom som har foranlediget Tvangs Anvendelse*).

2. Number of times on which restraint was had recourse to (*antal af Gange som Syge have været underkastede Tvang*).

3. Form of restraint (*Tvangsmidlets Beshaffenhed*).

4. Average duration of restraint (*gjennemsnitstid hvori Tvang er anvendt i de forskjellige Tilfælde*).

It is at least manly and frank to publish openly such a statement; it shows unmistakeably that the Norwegians are not victims to the hobby that all restraint — it matters not in what circumstances — is cruel, mischievous, and unnecessary. There are, perhaps, not many asylum superintendents in this country who, in the face of this well-known amiable and philanthropic, but withal crotchety, idea regarding the utter inadmissibility of the procedure or mode of treatment which is designated by that objectionable and ugly term *restraint*, would venture to publish such tables, or to defend their bold and independent practice. A reaction, however, appears slowly but surely to be taking place among those best conversant with the subject — among practical men, asylum superintendents — who begin to see the absurdity and fallacy of the supposed distinction between mechanical and personal restraint, and who find that cases will occur, notwithstanding the authority of certain very distinguished psychologists, necessitating some form of mechanical restraint, and, finding this, they act according to their convictions. It is surely monstrously absurd to call the application of a glove or belt "mechanical restraint," and to frown at seclusion as something horrible; while restraint by two or three attendants, who cannot possibly be expected to do more than human nature can—keep their tempers, under every amount and kind of irritation or provocation,—a restraint which implies, as every superintendent full well knows, black eyes, broken ribs, the most bitter antipathies, the most deadly struggles, the most inordinate excitement,—is denominated "moral suasion," or is called some equally pretty name! There can be no doubt, I think, that restraint by persons is in many cases infinitely worse than restraint by things; and I must confess that a suspicion attaches, in my own mind, to any system or any asylum professing to have no recourse whatever to "restraint." Undoubtedly, in all asylums it will be found in some form, so long as insanity and human nature remain what they are; or it will be supplanted by some equivalent mode of treatment. I have tried this system of supplying numerous attendants to destructive and violent patients, and I have frequently found it wanting. A superintendent must, of course, take attendants as they are, not as they ought to be. But, lest I should be misunderstood, it is necessary to state here that I have never had recourse to mechanical restraint in the sense in which the term is generally understood. Nevertheless, I can conceive cases to occur requiring it; and I should certainly not be deterred, if they did occur in my practice, by any dogmas or dominant ideas on the subject of non-restraint, from treating such cases by mechanical or personal restraint, or otherwise, as I should see fit. The replies of the English and Irish superintendents, to the Lunacy Commissioners of their respective countries, on this subject, are most manly and independent; but they show a remarkable discrepancy of opinion between the governing body, the theorists—and the governed, the practical men. I do not indulge in these remarks with a view to defend the use, in the Christiania State Asylum, of restraint or seclusion—far from it; for I am not in a position to say whether the restraint imposed was necessary at all, and, if so, whether it was of a proper kind and duration. I saw, during my visit, only one person under restraint, and that merely extended to the arms, so as to prevent any injury to a dislocated shoulder-joint, which had been set on the previous day. The general impression produced on my mind by what I saw was, that the superintendent was a most humane, judicious, experienced physician, who might safely be entrusted with the physical and moral, as well as medical, treatment of his patients. The symptoms, or conditions, on account of which restraint was prescribed in Gaustad, were the following:—Violence (*voldsomhed*), brutality (*brutalitet*), obstinacy (*opsætighed*), impertinence (*uartighed*), destructive tendency (*ödelæggelselyst*), dirty habits (*urenlighed*), self-mutilation (*selvbeskadigelse*), suicidal attempts (*selvmord-forsög*), fugitive tendency (*undvigningslyst*), onanism (*onani*), and restlessness (*uro*).* The forms of seclusion and restraint employed are—Seclusion (*indespærring*), the restraint-jacket, strait-waistcoat or camisole (*tvangströje*), the restraint-chair (*tvangsstol*), and the handcuffs (*haandböjler*). The duration varies greatly; some patients were secluded or restrained day and night, others by night only; some for sixteen hours at a time, others for fifty-five consecutive nights. The jacket was worn from one to fifteen days and nights (I give round numbers only), and "the chair" was occupied for from sixteen hours to three days and nights. It certainly would appear as if the mantle of the past had fallen on the Christiania State Asylum in regard to restraint and seclusion; but I have already said enough, I think, to indicate that the superintendent and the directors must not be judged hastily or harshly in regard to this department of their treatment or management. Dr. Sandberg himself declares that he expects restraint and seclusion to be gradually lessened in proportion as the staff of attendants is increased, and as better examples are set before new-comers by patients long resident. Such a declaration tends to show that he regards restraint and seclusion as mitigable evils, and that he intends diminishing them to a minimum amount or degree.

I come now to speak of the medical treatment of the patients.

* I give the Norwegian words, lest they should not be fully or correctly represented by my translation or interpretation thereof.

As the causes of insanity are psychical and physical, so does Dr. Sandberg divide the treatment into psychical and physical. He has evidently great confidence in the virtues of change of scene, removal from sources of irritation, abundance of fresh air, out-of-door exercise and occupation, fine view, good keep, and regular habits. But he has great faith, in addition, in bathing, as well as in opium, belladonna, digitalis, hyoscyamus, and other drugs. *Opium* appears to be his sheet-anchor in melancholia, where the circulation is languid, with a dry bluish skin, and rigid muscles. He uses it further in what many will be inclined to regard as heroic doses: he begins with 1 grain morning and evening, and goes up to 8 grains twice a day; not, however, in any case exceeding the latter dose. Sometimes he gives a grain every fourth, eighth, or tenth day only. Males are opiatised more fully than females, taking sometimes 4 to 8 grains twice per day, or on an average 2 to 7 grains morning and evening; while females begin at 1 grain, and gradually go up to 7, the average being 5 grains. Doses such as this were given in 24 cases—11 males and 13 females. No dangerous effects ever accrued, though headaches, nausea, and vomiting are specified as among the accidental effects of opium in individuals who appeared peculiarly susceptible to its action. The question of the tolerance by the system of opium in cases of mental disease, or in various severe physical diseases, was recently agitated in connexion with the death of Mr. Augustus Stafford, M.P.; and on other occasions, also, most curious and surprising evidence has been collected of the large quantities of opium, in some of its forms, that can be borne under certain circumstances of idiosyncrasy or disease. In many cases of mania, for example, ordinary doses of opium or of laudanum produce no effect. Several grains of opium or drachms of laudanum may be given with impunity; and persons become so habituated to its use, and so unsusceptible of its influence, that they can drink laudanum by the ounce, or even pint, as they would drink beer or wine! As adjuncts to opium, Dr. Sandberg uses, in some cases, the cold shower-bath or the warm-bath; the latter where the skin is cold or bluish. I cite a single and ordinary case of melancholia, as illustrative of the opium plan of treatment. It had been treated with infusion of digitalis, extract of stramonium, and subsequently 5 grains of opium daily, prior to admission. On 9th of July, the patient began to take opium in doses of 1 grain morning and evening; on 19th, the dose was increased to 2 grains twice a day; on 29th, to 3 grains; on August 7, to 5 grains; on 12th, an emetic powder; on 13th, continue opium, 5 grains, morning and evening; on 16th, 6 grains; on 28th, reduce to 5 grains; on September 7th, 4 grains; on October 1st, 3 grains; and on the 13th, discontinue the opium. Here is a patient taking opium for three months, almost daily, sometimes in doses of 12 grains per day—a mode of treatment of doubtful usefulness in such a case. Instead of opium itself, morphia was used—apparently the alkaloid itself, not its salts—in some 17 cases, 7 males and 10 females. This is exclusive of cases in which it was given as an ordinary and mere soporific. The average dose on commencement was $\frac{1}{4}$ grain morning and evening; subsequently, three times a day; and it was gradually increased to $\frac{1}{2}$ grain three times a day. Prolonged warm-baths, with a stream of cold water on the head, appear to stand in the same relation to mania that opium does to melancholia, in the Gaustad Asylum. The plan is precisely that now carried out successfully, and on the large scale, at the Royal Edinburgh Asylum, and other British asylums, the body of the patient, with the exception of the head, being immersed in warm water for some hours, while a stream of cold water constantly trickles over the head from a pipe suspended over it. This plan was had recourse to in 27 patients, 17 males and 10 females. It would appear from these statistics that, while severe cases of melancholia occurred chiefly among females, severe cases of mania were found chiefly among males. The duration of the bath system varied from one to eight hours per day, and consecutively; and as an adjuvant—a calmative—tartar emetic alone, or in conjunction with extract. belladonnæ, was sometimes employed. Seclusion in a darkened chamber seems also commonly resorted to in acute maniacal cases. Various forms of hydropathic treatment seem common in insanity in Norway, both within and without the Gaustad Asylum. Thus I find cases of stupidité (the acute dementia of some authors, and the melancholia, with stupor, of others) and of dementia treated with the warm or tepid-bath, and with the "*Regndusch*" and "*Gräfenbergerdusch*,"* in the open air. In addition to the medicaments or appliances I have named, Bavarian beer as a tonic; infus. digitalis as a sedative or calmative of the heart's action, when it is tumultuous; decoct. rhamni frang., ol. ricini, potass. bitart., flor. sulphur., infus. valerian., and various other drugs, appear to have a prominent place in the asylum pharmacopœia. Sulphate of copper was used in 4 males—cases of maniacal paroxysm; digitalis, with or without nitre, in 3 males and 1 female; quinine and decoct. chinæ acidum, in 2 males and 1 female; camphor, in 2 males and 3 females, cases of erotomania, in doses of 1 to 4 grains three times a day. As tonics and emmenagogues, pills of rhubarb and ext. conii, pil. tonico-nervin, tinct. mur. ferri, infus. rheorum, &c., were employed; and, in other special indications, atropin, fibrill. rad. artemisiæ vulg. solut., saccharated iodide of iron, iodide of

* The Norwegian "tvangsdusch" seems to be our old douche proper, used more for purposes of punishment and terror than as a tonic!

potassium, electuar. anthelmint., emulsio assafœt., liquor antispasmodic, &c. Derivation by purgation was effected by decoct. rhamni frang., solut. salis anglici, ext. rhei comp., &c. Counter-irritation was applied in the form of antimonial (tartar emetic) ointment (*stibiatsalve*) and of vesicatives to head or nape of neck. The spontaneous derivation or counter-irritation effected by boils and carbuncles was sometimes most salutary. A great portion of Dr. Sandberg's valuable Report consists of most interesting details of individual cases of the various forms of insanity, according to the classification, which he recognises, with their symptomatology, pathology, and treatment; but for these I must refer the reader to the Report itself.*

Of a far different character, in regard, at least, to site, construction, and general arrangements, is the Christiania City Asylum at Mangelsgaarden, buried as it is in one of the town thoroughfares. I was accompanied in my visit both by the consulting and resident physicians, Drs. Winge and Kaiser; but I take the information I have to give regarding it chiefly from a most interesting pamphlet by the former gentleman,—a reprint from the "Norwegian Journal of Medicine,"—which pamphlet consists, in great measure, of a series of cases, with commentaries as to the psychical phenomena and medical treatment.† Like the Gaustad Asylum, this institution appears to date from the law of the Storthing regarding lunacy and lunatic asylums, dated 17th August, 1848: a law and a date which seem to have constituted an era in the history of insanity and asylums in Norway. From this law will undoubtedly date still further changes in the number and character of Norwegian asylums. So soon as the cautious and shrewd Norwegians see the practical working of the present expensively constructed and organized State Asylum, and thereby

* I would strongly advise all intending medical visitors to Norway and its asylums to "rub up" their German or French if they are not acquainted with Norwegian. I found many of the best-educated classes in Norway—professors in the University, and the merchants and citizens of Christiania—ignorant of the English language. German is the foreign language chiefly spoken by the educated Norwegians; next to it stands French; while English is only beginning to be generally cultivated as a language. Dr. Sandberg, for instance, does not speak English; but he spoke to me fluently in German. Dr. Winge, Physician-in-Chief, and Dr. Kaiser, Assistant or Resident Physician of the Christiania City Asylum, however, both speak English comparatively well, especially the latter. To thoroughly enjoy and appreciate a tour in Norway, and to derive material advantage therefrom, it is extremely advisable, if not absolutely necessary, that the traveller should know something of either the Norwegian or Danish languages. The latter is comparatively easy of acquisition, and there is no difficulty in procuring grammars, dictionaries, and other linguistic aids. If he do not do this, his tour will either be a series of misadventures and disagreeables, or he must place himself in the hands of guides or couriers—both an expensive and irksome procedure.

† "Beretning om Christiania Sindssyge-asyls Virksomhed i Aarene 1850—1856. Af P. Winge, Overlæge. (Copie af Norsk Magazin for Lægevidenskaben.) Christiania. 1857."

discover its demerits as well as recognise its merits, others, on a smaller and less pretentious scale, will probably be built in different parts of the country. For if the necessity for numerous small district asylums is keenly felt in Scotland, which is comparatively well furnished with water, if not land, conveyance, it must be felt still more seriously in a primitive country like Norway, where land conveyance, in particular, is extremely slow and imperfect.

The Christiania City Asylum resembles externally some of our older Scotch poor-houses. It is separated from the public road or street only by a dingy court, with an appearance not at all prepossessing. Nor are the internal architectural arrangements specially commendable. The most, however, has evidently been made, by the intelligent physicians, of the accommodation and materials at their disposal; the general principles of management or treatment being precisely those to be found in operation at Gaustad, and which I have already detailed. The building was originally a custom-house or poor-house, or was devoted to some such public use: it was partly converted into an asylum in 1849, and entirely so in 1851. It accommodates only 60 patients, 30 of either sex. It is intended mainly for paupers; but private patients are admissible if there is room, and the cases are considered otherwise suitable. All the larger or principal rooms look to the south and east; but there is no view, so far as I remember, from any of the lower windows—at least, except brick walls and blocks of houses! The rooms, of whatever size, have a height of 5 ells—that is, 10 feet, the Norwegian ell being equal to about 2 feet English. There is on the male side of the house a wing or department for the quiet and cleanly, and another for the violent and dirty. A similar arrangement exists on the female side; but the building does not pretend to symmetry, nor to architectural facilities for classification. The male wing for the quiet and cleanly contains a large parlour (*forsamlingsværelse*); a large dormitory (*sovesal*), fitted up with 11 or 12 beds, and a smaller one with 7 or 8 beds; a dining-room (*spisestue*); and workshops for carpentry, turning, basketmaking, shoemaking, and other useful handicrafts. The male wing for the violent and dirty contains 8 bed-rooms, each 8 ells deep, or 16 feet, 5 ells broad, and 5 high. In some of them the windows are guarded by a row of palisades, at a distance of 1½ ells, or 3 feet. These rooms struck me as being dark, damp, and dingy; and the same objectionable arrangement exists as at Gaustad in regard to night-stools in a corner of the room. They are furnished, as are also the bath-rooms, with floors of asphalte about ¾ inch deep. The male side of the house also possesses attendants'-rooms, wardrobe-rooms or store-rooms for clothes, and bath-rooms, with antechambers for dressing and undressing, and other conveniences. The bath-rooms are very neatly and comfortably furnished, and fitted up with plunge and shower-baths; but they are inconveniently small and dark. The water-closets likewise possess the latter objection. The corridors are narrow and dark, the single rooms small and cell-like; the airing courts contracted and gloomy, with little vegetation; and altogether there is a poor-house aspect and feeling about the place. These, however, are all faults inseparable from the reconstruction or adaptation of an old-fashioned house, and for which the medical officers are nowise responsible.

The female wing for the quiet and cleanly has a large association-room (*forsamlingssal*), where the patients busy themselves in needlework and other suitable occupations during the day; a large dormitory (*sovesal*) with 14, and another with 10 beds; and a roomy kitchen, which serves also as a dining-parlour. The use thus made of the kitchen has the advantage that the patients themselves superintend the cooking of their meals, and the washing and placing of their dishes; and hence, an arrangement that has undoubtedly been necessitated by want of room, is really productive of the best results, in so far as homely habits are engendered and profitable occupations taught. The kitchen, however, is sunk a few feet below the ground, and the entrance is by sunk steps. This is a bad arrangement, and is only defensible on the score of the age of the building and its originally bad construction. The female department for the violent and dirty contains 6 commodious single rooms,—I detest the word "cells," although the Norwegians themselves employ the term "*celle-værelser*,"—lighted from the roof (by skylights), with rooms for attendants, and wardrobe-rooms, as on the male side. The whole establishment is lighted with gas, and seems sufficiently well supplied with water. From the small size of the whole building, and the compactness of the rooms, the inmates have much of a family character in their habits and occupations. Indeed, such an arrangement is a comparatively close approximation to the colony system of Gheel,—the cottage mode of treatment of some English and Scotch asylums. It is doubtful, without actual experiment, to what extent this system of treatment can be carried out in this country, modified, as it necessarily would be, according to the class and kind of patients, the rates of board, the site of the colony, and its proximity to towns. But I am firmly of opinion that the principle on which such system is based is the proper one, though I must own to seeing many obstacles in the way of having it practically carried out at present in this country. One great obstacle is, at the outstart, its expensiveness; and this is a terrible bugbear and barrier to progress in the eyes of ratepayers; there is little prospect, therefore, of any such plan being voluntarily carried out extensively or fairly in purely pauper asylums. Economy is in favour of large and compact asylums, which are said, or supposed, to be the cheapest means of providing, according to Government regulations, for the insane. Humanity and science recommend small asylums, as certainly affording the best chances of cure and comfort; and on the Lunacy Commissioners is forced the difficult and delicate task of reconciling parties holding such opposite views, and of indicating some middle course satisfactory to both.

The staff of this asylum appears lamentably meagre. On the male side, there are a head-attendant and 2 ordinary attendants; and on the female side, a matron and 2 female attendants. The following Table shows the statistics of the institution in regard to the admissions and discharges during the six years preceding 1857:—

*Table showing the Admissions and Discharges in the Christiania City Asylum (at Mangelsgaarden), between* 1850 *and* 1856.

| Year. | Admissions. | | Discharges. | | | | | | | |
|---|---|---|---|---|---|---|---|---|---|---|
| | | | Cured. | | Improved. | | Unim-proved. | | Dead. | |
| | M. | F. | M. | F. | M. | F. | M. | F. | M. | F. |
| Remaining in 1849 | 22 | — | — | — | — | — | — | — | — | — |
| Admitted in 1850 | 33 | 5 | 13 | 2 | 5 | — | 5 | 1 | 4 | — |
| „ 1851 | 27 | 20 | 10 | 2 | 7 | 1 | 4 | — | 6 | — |
| „ 1852 | 20 | 27 | 6 | 7 | 3 | 3 | 4 | 4 | 6 | 2 |
| „ 1853 | 29 | 21 | 10 | 11 | 5 | 4 | 4 | 4 | 5 | 4 |
| „ 1854 | 12 | 20 | 7 | 5 | 2 | 3 | 3 | 5 | 4 | 5 |
| „ 1855 | 12 | 18 | 3 | 11 | 2 | 3 | 7 | 3 | 1 | 3 |
| „ 1856 | 14 | 16 | 4 | 8 | 1 | 5 | 3 | 2 | 3 | 4 |
| Total . . . . | 169 | 127 | 53 | 46 | 25 | 19 | 30 | 19 | 29 | 18 |

Remained under treatment at close of year 1856, 57 patients—32 men and 25 women.

Of the total number of 296 patients under treatment between 1850 and 1856, there were—

| | | | |
|---|---|---|---|
| Cured . . . . . . | 99, | or about 33⅓ | per cent. |
| Improved . . . . . | 44, | „ 17 | „ |
| Unimproved . . . | 49, | „ 17 | „ |
| Dead . . . . . . | 47, | „ 17 | „ |

The dietary regulations, like those of Gaustad, are sanctioned by the "Control Commission," or Lunacy Board; but they differ somewhat from those of that asylum in detail. I have presented them in a tabular form. For full diet, a charge of 17 skillings is made for each patient per day—that is, about 8½*d.* English.

*Table showing the Dietary Regulations in the Christiania City Asylum (Mangelsgaarden).**

| Sunday. | Monday. | Tuesday. | Wednesday. | Thursday. | Friday. | Saturday. |
|---|---|---|---|---|---|---|
| | | | 1. *Morning (Breakfast).* | | | |
| $\frac{1}{2}$ quart tea, $\frac{1}{4}$ oz. sugar, $\frac{1}{2}$ lb. bread, and $\frac{1}{2}$ oz. butter. | As on Sunday. | As on Sunday. | As on Sunday. | As on Sunday. | As on Sunday. | As on Sunday. |
| | | | 2. *Mid-day (Dinner).* | | | |
| 1 quart meat soup, with peas, groats, and potatoes; 4 oz. salt beef, and $\frac{1}{4}$ lb. bread: or 1 quart meat soup, with groats or vegetables; 4 oz. fresh beef, and $\frac{1}{4}$ lb. bread. | 1 quart öllebröd, 1 pint lapskous, and $\frac{1}{4}$ lb. bread. | 1 quart meat soup, with groats and vegetables; 4 oz. fresh beef, and $\frac{1}{4}$ lb. bread. | 1 quart gryn-gröd and 1 pint sweet beer: or $\frac{3}{8}$ quart milk, $\frac{1}{4}$ lb. bread, and $\frac{1}{4}$ oz. butter. | As on Tuesday. | 1 quart milk broth, with barley groats; 1 portion salt fish or fresh fish, and $\frac{1}{4}$ lb. bread.† | 1 quart öllebröd, and either 1 pint lapskous and $\frac{1}{4}$ lb. bread, or $\frac{1}{4}$ lb. bread, $\frac{1}{4}$ oz. butter, and 1 portion salt herring. |
| | | | 3. *Afternoon Lunch (Eftermiddag).* | | | |
| $\frac{1}{4}$ lb. bread and butter. | As on Sunday. | As on Sunday. | As on Sunday. | As on Sunday. | As on Sunday. | As on Sunday. |
| | | | 4. *Evening (Supper).* | | | |
| $\frac{1}{4}$ lb. bread, $\frac{1}{4}$ oz. butter, and 1 pint beer. | $\frac{3}{4}$ quart gryn-gröd and 1 pint beer, with $\frac{1}{2}$ oz. sugar and $\frac{3}{8}$ quart milk: or, in option of Superintendent, an allowance of butter and bread, or milk and bread. | As on Monday. | As on Sunday. | As on Monday. | As on Monday. | As on Monday. |

For explanation of the terms used in the foregoing Table, and of the composition of the dishes, &c., see Table of the Dietary in the State Asylum at Gaustad, p. 269 of this paper.

* Milk, barley-broth (byg-suppe), white bread, and tobacco, are regarded as luxuries or extras, for which an additional charge is made.

† To fish diet on Fridays, potatoes if in stock; in which case, the allowance of bread is diminished to one-half.

Relapses occurred once in 16 cases, twice in 3. They happened in 2 cases after an interval of 2 months subsequent to discharge; in 2 after 6 months; in 1 after 7 months; and in 2 after 10 months; while, in the remainder, the interval was from 1 to 3¾ years after apparent recovery and discharge from the asylum.

Here, as at Gaustad, the importance of occupation is fully recognised, and a large proportion of the patients is engaged in useful labour. The kinds of work chiefly engaged in are as follows:—Basket-work and wicker-work (*kurvbinderarbeide*), carpentry or joiners'-work (*snedkerarbeide*), tailor-work (*skræderarbeide*), shoemaking (*skomagerarbeide*), glovemaking (*handskemagerarbeide*), wool-teasing, &c. (*uldplukning*), carding and spinning wool (*karding og spinding*), knitting and ribbon-weaving (*strikning og baandvvæning*), sewing (*söm*), and household-work, &c. (*huustjeneste og udvendigt arbeide*). Dr. Winge does not hesitate to avow the use of restraint and seclusion, with a view to prevent patients hurting either themselves or others, or destroying the property of the asylum. Not only is the strait-jacket used as a means of treatment, but seclusion, with or without the cold shower-bath, is resorted to as a means of correction or punishment. I quote the paragraph in Dr. Winge's pamphlet relating to restraint and punishment, as one of some importance, showing the different light in which both are regarded in Norway, as compared with this country:—

"Hvad Tvangsmidlernes Beskaffenhed og Anvendelse angaaer, da indskrænkes Brugen af dem til at den Syge, saavidt muligt, hindres fra at tilföie sig selv, andre Personer, eller Asylets Eiendom nogen Skade. De bestaae i Afsöndring i Eneværelse og Anvendelse af Tvangströie. Som Correctionsmiddel anvendes undertiden Eneværelse, undertiden, og i Almindelighed da med udmærket Virkning, et koldt Styrtebad."*

In the same pamphlet, Dr. Winge divides mental diseases into the following classes:—

A. Those induced by, or depending upon, a primary derangement of the nervous system:—
  - *a.* Depending on a primary lesion of brain. (p. 12.)
  - *b.* Depending on a primary lesion of spinal cord. (p. 32.)
  - *c.* Depending on a primary lesion of eccentric nerve-system. (p. 39.)

B. Those induced by, or depending upon, a primary derangement of the circulation or blood. (p. 43.)

C. Those induced by, or depending upon, a primary derangement of the sexual system. (p. 50.)

D. Those induced by, or depending upon, a primary derangement of the digestive apparatus. (p. 63.)

* "Beretning om Christiania Sindssygeasyls Virksomhed i Aarene 1850—1856," p. 8.

A section at the end of Dr. Winge's paper is devoted to the medical treatment of the cases in the Christiania City Asylum. He has evidently great faith in medicinal treatment; and he uses some powerful remedies with no sparing hand. From *opium* he has frequently seen the most rapid and remarkable effects, when given in large doses. He considers it as indicated in cases of great cerebral irritation, or inordinate nervous excitement, or where precordial pains, sleeplessness, and other distressing symptoms exist. It is sometimes used in the form of powder, with sugar, in one or several doses per day. He generally begins with 1-3 grains morning and evening, increasing the dose by 1 grain every fourth or fifth day, until it reaches 6-8 grains, or even 14 grains, when it is gradually diminished every third or fourth day, until the patient is again taking only 1 grain at a time. He declares the fear to be a groundless one, that constipation, poisoning, &c., will infallibly follow such large doses of opium. He has seen vomiting induced by medium doses—4 to 5 grains; but this symptom has disappeared on increasing or diminishing the dose, according to circumstances. He has never seen any subsequent dangerous depression attributable to opium. It is given cautiously, and in small doses, if at all, where there is a torpidity of system, a tendency to stagnation in the portal system, disease of heart, chest, or abdomen, or symptoms of general paralysis. It is sometimes combined with iron, quinine, and other tonics. Dr. Winge does not regard cerebral congestion or mental depression as contra-indications; for he has seen the greatest benefit from its use in both these cases. Instead of small doses of opium, where stronger narcotism is contra-indicated—where depression exists with hypochondria and weak pulse — *acetate of morphia* is sometimes used, either in the evening in doses of $\frac{1}{4}$ to 1 grain, or 2 or 3 times a day in smaller doses. In cases of organic disease of the heart, or of increased or tumultuous action thereof, especially where a sub-inflammatory or exudative process is supposed to be going on in the meninges, *digitalis* is given. The form of administration is *acetum* digitalis, in doses of 10-30 drops, 3 or 4 times a day; or as infusion (gr. xv.—ʒi. to ℥viii.), 1 tablespoonful every 2, 3, or 4 hours. Infus. digitalis with borax is also recommended as a good emmenagogue. *Conia* has been used in some cases to reduce a rapid pulse, to diminish increased action of the heart, or to subdue cardiac irritation:—gr. i., dissolved in ʒii. aq. fl. naphæ, of which 3-8 drops were given 3 or 4 times a day. Dr. Winge's experience of this remedy is, he says, too limited to enable him to give any decisive opinion as to its merits. *Stramonium* has been used with success in cases of hallucination of hearing, either in the form of pill—2-10 gr. of extract. stramonii per day, in one dose at bedtime, or in smaller quantities morning and evening,—or in that of solution in water. Under its use, Dr. Winge has several times seen dilatation of the pupil and impairment of vision. *Haschisch,* or extract. cannabis indicæ, is frequently used in melancholia accompanied by stupor, or an adynamic state of system. It is given in small doses—gr. ½-2, once or twice a day, and always in the form of pill, on account of its taste. Its effect is generally most exhilarating and enlivening. Oxide of zinc (flores zinci) is occasionally used in convulsive affections, with or without small doses of opium. *Valerianate of zinc* has been employed in acute cases, where nervous phenomena were prominent, and the patient was noisy and excited. After its use, opium has sometimes been found more efficacious. The difficult subject of *bleeding* in insanity is slightly touched upon. It has been resorted to in a few exceptional cases; and a moderate bleeding is stated to be useful in incipient meningitis, where there is absolute plethora, or an engorgement of the vessels of the brain. Cases therefore occur in which it is decidedly indicated. But it is not generally recommended; on the contrary, Dr. Winge denounces it as usually most hurtful—often inducing collapse, or an incurable form of insanity. For producing derivation of blood from the head, and to promote menstruation, *Junod's boot* is spoken of in a commendatory way. Dr. Winge has never seen secondary cerebral congestion, or dangerous reaction—cerebral or local—result from its use. It is usually applied to one limb at a time; so that if it be placed on the right leg or arm to-day, it is applied to the left to-morrow; or it may be applied on the same day, for a short time, first to the one limb and then to the other. Sometimes, however, it was borne so well by the patient, that it could be applied to both extremities at once. *Warm* and *tepid* baths are commended as not only in themselves good, tending to produce sleep, subdue erethism, and restore and increase the action of the skin, but as most useful in assisting the action of medicinal remedies. The *prolonged warm-bath, with cold douche to the head,* is likewise extensively employed, after the manner of Gaustad. Many of the foregoing remedies, medicinal or otherwise, have been tried experimentally only, and for comparatively short periods; but their bare enumeration suffices to indicate the enterprising and enlightened spirit which guides the practice of the psychologists of Norway.

Let us now briefly glance at the lunacy laws of Norway, the Government machinery for the treatment of the insane and the regulation of asylums; and thereafter let us consider the statistics of insanity in Norway, according to the census of 1845, with a view specially to ascertain the extent to which proper accommodation—that is, public asylum accommodation—has been provided for her insane population. The law of the Storthing—which is also the imperial law, having received the imperial fiat or imprimatur—relating to lunacy, is a modest 4to pamphlet of some five pages, bearing the signature of Oscar, King of Norway and Sweden, and dated at Malmö, 17th August, 1848.* It consists of but five short, concise chapters, divided into twenty-one sections. The preamble narrates that "Vi Oscar af Guds Raade Konge til Norge og Sverige, de Gothers og Venders," proclaim by these presents that we have had laid before us the statute or decree of the Storthing,† of the 11th of July of this year (1848); and the Royal proclamation winds up by adopting and confirming, like as we do hereby adopt and confirm, this statute or decree henceforth as the law of the land. The first chapter is devoted to the subject of the regulations anent the construction and management of asylums (*om Sindssygeasylers Oprettelse og Bestyrelse*). There are eight sections, which I cannot, of course, here embody at full length,—nor is this necessary. Section 1 provides that all asylums must be authorized by Royal sanction; and, with a view hereto, it requires that there should be submitted to Government full plans of the buildings and grounds of all proposed asylums, with estimates thereanent. The petitioners must exhibit the details of their scheme of management, including, for instance, the list of officers, and their proportion to the number of patients; the pauper dietary, the industrial resources, the means of social recreation, of classification, of restraint, the provisions for cleanliness, &c. It enacts that an asylum must have no connexion with other institutions of any kind, that the site must be salubrious, the provision for occupation and exercise abundant, and that there shall be a due separation of the sexes, and a proper classification of the patients, according to the phase or form of insanity or otherwise. Section 2 relates to the licensing or authorization of private asylums. Section 3 refers to the medical officers of asylums, who must be duly qualified by law. Section 4 admonishes to social harmony in asylums, and advises constant employment; recommends that the application of seclusion and mechanical restraint should be as seldom as possible—only when absolutely necessary; enacts that there shall be no corporal punishment (*legemlig Revselse maa ikke finde Sted*). Section 5 enacts that in every asylum be kept a "Personal Protocol," or register of patients, which will

* "Lov om Sindssyges Behandling og Forpleining."

† The "*Storthing*," or Norwegian Parliament, meets every three years; it consists of the "*Lagthing*," or Upper House, and the "*Odelsthing*," or Common House. Measures passed by it require the Royal sanction before they can become law, unless they pass through both divisions of the house in three successive Storthings.

contain the full names of each patient, with his or her age, birthplace, and occupation, as well as the name of the party at whose instance or request he or she was admitted; and a "Behandlings Protocol," or register of treatment, which shall contain the name of every patient placed in seclusion or under mechanical restraint, with the grounds for such treatment and its duration, and shall also exhibit the number of patients daily employed, with the nature of such occupation. The "Personal Protocol" shall further contain, within eight days after the admission of each patient, an account of his or her physical and mental state; the date of deaths, with the causes thereof; the date of discharges or removals, with the names of the persons at whose instance such removals or discharges have taken place; the grounds or causes thereof; the condition of the patient on removal; and, so far as possible, his or her future residence. The protocols or registers—which the reader will at once recognise to be almost identical with the asylum registers in this country—are ordered to be laid before, or exhibited to, the Commissioners of Control (*Controlcommissairerne*), or Commissioners in Lunacy, as we call them in this country, at every visit, by whom they will be examined, and thereafter subscribed with any remarks regarding the entries which they deem it necessary or advisable to make. Section 6 provides that the superintendent or physician to every asylum shall, every three months, send extracts from the protocols mentioned in the preceding section, with a copy of the remarks of the Commissioners therein, as well as an Annual Report of the general condition of the establishment under his charge, to the Board of Control (Controlcommission). Section 7 enacts that control over each asylum be vested in a body of Commissioners, who must reside in the neighbourhood, so as to be at all times accessible; this board to consist of three members, of whom one must be a qualified medical man, appointed by the King. A special code of instructions is drawn up by Government for their guidance.* Every asylum may, in addition, be inspected by any person specially appointed for that purpose by the King, at such times and in such manner as he, the King, sees fit. Section 8 relates to the period when the act or law comes into operation.

Chapter II. relates to the admission of patients (*om Sindssyges Optagelse i Asyler*). Section 9 empowers the superintendent or physician of each asylum to inquire into the advisability or

* "Instrux for Controlcommissioner i Overeenstemmelse med Lov om Sindssyges Behandling og Forpleining af 17de August, 1848."
"Instructions for the Commissioners of Control, in accordance with the Law of 17th August, 1848, relating to the Maintenance and Treatment of the Insane."

necessity of admitting each applicant, either on the ground of his own mental state, or for the safety and security of the public, and to decide thereanent, it being provided that in case of such applicant or his representatives being dissatisfied with the decision of such superintendent or physician, he or they may appeal to, and must abide by, the deliverance of the Board of Control. Section 10 defines the duty of the police in regard to insane persons committing breaches of the public peace, or who have no relatives or parties responsible for their conduct or maintenance. Section 11 provides that a report of each admission, extracted from the "Personal Protocol" before referred to, along with an account of the patient's condition, be sent to the Board of Control within forty-eight hours after such admission. The board shall, immediately in cases of complaint or under unusual circumstances of any kind, or, in ordinary cases, at their next visit to the asylum, institute such inquiries as will enable them to decide whether the patient is a fit subject for confinement or not.

Chapter III. relates to the discharge of patients (*om Sindssyges Udtrædelse af Asyler*). Section 12 sanctions the discharge, by the superintendent or physician, of recovered cases. Section 13. The discharge of patients uncured (improved only, or unimproved) is to a certain extent at the option of their relatives or guardians—that is, provided they have not been placed in the asylum at the instance of the legal authorities, or the removal proposed is not, in the opinion of the superintendent or physician, fraught with danger either to the patient or to the public—in which latter cases the decision of the Board of Control is final and binding. Section 14. Discharges and deaths of all patients are to be reported, along with an account of the condition of each patient at the time of such discharge or death, by the superintendent or physician, both to the Board of Control and to the relatives or guardians,—to the former within forty-eight hours, and to the latter as soon as possible after such event.

Chapter IV. relates to patients living in or with their own families, or in private houses or asylums (*om Sindssyge der forblive hos deres Familie, eller udsættes i privat Forpleining*). Section 15 renders it compulsory to report without delay, to a qualified medical man, either directly or through the clergymen of the parish or district, every case of insanity, or supposed insanity, said medical man being in such event called upon forthwith to examine and report as to the nature of the case, the expediency and form of confinement, &c. Section 16 renders it obligatory to place all violent patients in asylums. It is the duty of medical men to report to the legal authorities all cases of doubtful insanity which may come to their knowledge in private practice. Section 17 places harmless insane paupers, who, from the nature of their insanity, do not require special treatment or confinement, on the footing of ordinary paupers in regard to maintenance, &c. Section 18 obliges medical men to report at the end of every year to the "Medicinal-bestyrelse"—apparently a sort of supreme national medical board—the names and condition of their insane patients.

Chapter V. consists of general regulations (*almindelige Bestemmelser*). Section 19 relates to the source of payment of board for paupers, and the allocation of the expense of their maintenance in asylums. The sum is to be assessed on the native district or birthplace in certain events. Section 20 enacts that no insane patient be kept along with criminals. Section 21 establishes fines and punishment for neglect or infringement of any of the foregoing laws or regulations, with withdrawal of licence in the case of private asylums or houses, &c.

It is scarcely necessary to call attention to the stringent tendency of the enactments of the Norwegian Law of Lunacy of 1848; but it may not be amiss to refer specially, as a contrast to the bulky, confusing, contradictory, and most unsatisfactory lunacy laws of England and Scotland, to its extreme shortness, conciseness, and simplicity. In connexion with the Norwegian law, as briefly here expounded, I have yet to advert to the powers of the Board of Control, as given in their special code of instructions from Government already quoted. The Commissioners of Control are entrusted with the supervision of the treatment of the patients, and the economy of the establishment, in all asylums. They lay down both general rules applicable to all asylums, and special rules for individual establishments. They visit each asylum twice a month, investigating all matters falling within their jurisdiction; but they may visit, or any individual member of the Board of Control may visit, whenever they or he shall see fit or necessary; they may examine particularly into the nature and amount of work done by patients, of their social pleasures and creature comforts, into the kind, frequency, and duration of restraint, the nature of the dietary, the condition of instruction and religion, the due classification of patients, &c. They receive and inquire into all complaints from patients or attendants, and they determine the kind and amount of punishment for offences. They also receive and inquire into complaints or statements in reference both to the admission and discharge of patients; in cases of differences of opinion between the superintendents or physicians of asylums, and the relatives or guardians of patients; or in cases of appeal against the decision or procedure of such medical officers. They make entries in the asylum registers of all matters falling under their observation which may seem to them calling for, or worthy of, record. They must account for all instances of absence from duty on their own part, and make a marking of the causes thereof in the asylum registers. Their powers and privileges appear to be otherwise very similar to those of our Commissioners in Lunacy in this country, except that they are more special and minute. I think I have said enough to prove that the Norwegians are by no means behind ourselves in respect of a liberal, humane, and enlightened treatment of their insane.

I must now draw my remarks to a close, by a few considerations on the statistics of insanity in Norway; and here I have to acknowledge my obligations to the several publications of Professor Holst, of the University of Christiania, on this subject, as well as to that gentleman individually, for much valuable information regarding the insane and asylums in Norway, communicated in the course of conversation. He has published the statistics of insanity as gathered from three several censuses of Norway—viz., those of 1825-6, 1835, and 1845.*

The latest statistics to which I have had access are those founded on the census of 1845, as contained in a paper published by Professor Holst in the "Norwegian Journal of Medicine," and kindly given me for the purpose of reference by the Professor himself.† In order to ensure accurate information, I give his own tables and remarks unaltered, except so far as translation is concerned. His paper includes the blind, deaf and dumb, and leprous; but with these we have here nothing to do, and I have accordingly omitted all notice of them.

* His investigations will be found in—

1. "Gersons and Julius' Magazin d. ausländischen Literatur d. gesammten Heilkunde. 1828. July—August, s. 10—23."

2. "Annales d'hygiène publique et de Médécine légale." Paris. 1830. Tom. iv. s. 332—359.

3. "Allg. Zeitschrift f. Psychiatrie und psych.-gerichtl. Medicin. 4 Band. 1847. s. 479—487."

In his statistics, deduced from the first two censuses, those of 1825-6 and 1835, he adopts the classification of the forms of insanity employed by Pinel and Esquirol —viz., a division into the great types of mania, melancholia (monomania), dementia, and idiocy.

† "Sindssyge, Blinde, Dövstumme og Spedalske i Norge den 31 Dec., 1845. Ved Prof. Dr. Frederik Holst, Aftrykt fra Norsk Magazin for Lægevidenskaben, V. Bd. 1851."

"Statistics of the Insane, Blind, Deaf and Dumb, and Leprous in Norway, according to the Census of 31st December, 1845."

I.—*Table showing the Number of Insane in Towns in Norway, according to the Census of* 31*st December*, 1845.

| Names of Towns. | Furious.* | | | Fatuous.† Congenital. | | Fatuous.† Subsequent to Birth. | | Fatuous.† Congenital and Acquired. | | | Furious and Fatuous. | | | Population. | | | Proportion of Insane to Population. |
|---|---|---|---|---|---|---|---|---|---|---|---|---|---|---|---|---|---|
| | M. | F. | Total. | M. | F. | M. | F. | M. | F. | Total. | M. | F. | Total. | Males. | Females. | Total. | |
| Christiania | 11 | 9 | 20 | 1 | 1 | 1 | 1 | 2 | 2 | 4 | 13 | 11 | 24 | 16,109 | 15,594 | 31,703 | 1:1321 |
| Dröbak | 1 | 1 | 2 | ... | 1 | ... | ... | ... | 1 | 1 | 1 | 2 | 3 | 675 | 675 | 1,350 | 1:450 |
| Hvidsteen | ... | ... | ... | ... | ... | ... | 1 | ... | 1 | 1 | ... | 1 | 1 | 41 | 59 | 100 | 1:100 |
| Hölen | ... | ... | ... | ... | ... | ... | ... | ... | ... | ... | ... | ... | ... | 100 | 99 | 199 | — |
| Soon | ... | ... | ... | ... | ... | 2 | 1 | 2 | 1 | 3 | 2 | 1 | 3 | 189 | 207 | 396 | 1:132 |
| Moss | ... | 1 | 1 | ... | ... | ... | ... | ... | ... | ... | ... | 1 | 1 | 1,927 | 2,096 | 4,023 | 1:4023 |
| Sarpsborg | ... | ... | ... | ... | ... | ... | ... | ... | ... | ... | ... | ... | ... | 653 | 672 | 1,325 | — |
| Frederiksstad | ... | ... | ... | 3 | ... | ... | 1 | 3 | 1 | 4 | 3 | 1 | 4 | 1,393 | 1,323 | 2,716 | 1:679 |
| Frederikshald | 2 | 2 | 4 | ... | 2 | 2 | 4 | 2 | 6 | 8 | 4 | 8 | 12 | 2,788 | 3,222 | 5,790 | 1:482·5 |
| Lillehammer | ... | ... | ... | ... | ... | ... | ... | ... | ... | ... | ... | ... | ... | 354 | 341 | 695 | — |
| Drammen | 3 | 2 | 5 | 7 | 2 | 5 | 7 | 12 | 9 | 21 | 15 | 11 | 26 | 3,910 | 4,466 | 8,376 | 1:322·2 |
| Holmsboe | 1 | ... | 1 | ... | ... | ... | ... | ... | ... | ... | 1 | ... | 1 | 148 | 145 | 293 | 1:293 |
| Svelvigen | ... | 1 | 1 | ... | ... | ... | 1 | ... | 1 | 1 | ... | 2 | 2 | 559 | 642 | 1,201 | 1:600·5 |
| Kongsberg | 1 | 5 | 6 | 1 | 3 | 1 | ... | 2 | 3 | 5 | 3 | 8 | 11 | 1,996 | 2,140 | 4,136 | 1:376 |
| Holmestrand | ... | ... | ... | ... | ... | ... | ... | ... | ... | ... | ... | ... | ... | 817 | 891 | 1,708 | — |
| Aasgaardstrand | ... | ... | ... | ... | ... | ... | ... | ... | ... | ... | ... | ... | ... | 216 | 228 | 444 | — |
| Tönsberg | 2 | 1 | 3 | ... | ... | 2 | 3 | 2 | 3 | 5 | 4 | 4 | 8 | 1,041 | 1,204 | 2,245 | 1:280·6 |
| Sandefjord | ... | ... | ... | ... | 1 | ... | ... | ... | 1 | 1 | ... | 1 | 1 | 336 | 428 | 794 | 1:794 |
| Laurvig | ... | ... | ... | 2 | ... | 6 | 3 | 8 | 3 | 11 | 8 | 3 | 11 | 1,886 | 2,126 | 4,012 | 1:364·7 |
| Skien | ... | 2 | 2 | ... | 1 | 2 | 1 | 2 | 2 | 4 | 2 | 4 | 6 | 1,740 | 1,937 | 3,677 | 1:613 |
| Porsgrund | ... | ... | ... | 3 | 1 | 1 | 2 | 4 | 3 | 7 | 4 | 3 | 7 | 1,043 | 1,171 | 2,214 | 1:316·3 |
| Brevig | ... | 2 | 2 | ... | ... | ... | ... | ... | ... | ... | ... | 2 | 2 | 730 | 725 | 1,455 | 1:727·5 |
| Stathelle | ... | ... | .. | 1 | 1 | ... | ... | 1 | 1 | 2 | 1 | 1 | 2 | 160 | 159 | 319 | 1:159·5 |
| Langesund | ... | ... | ... | ... | ... | ... | 1 | ... | 1 | 1 | ... | 1 | 1 | 304 | 323 | 627 | 1:627 |
| Krageröe | ... | 1 | 1 | 2 | ... | ... | 2 | 2 | 2 | 4 | 2 | 3 | 5 | 1,286 | 1,454 | 2,740 | 1:548 |
| Österüsöer | ... | ... | ... | ... | ... | ... | 3 | ... | 3 | 3 | ... | 3 | 3 | 942 | 1,066 | 2,008 | 1:669·3 |
| Tvedestrand | ... | ... | ... | ... | ... | 2 | 3 | 2 | 3 | 5 | 2 | 3 | 5 | 226 | 236 | 462 | 1:92·4 |

TABLE I.—*continued.*

| Names of Towns. | Furious.* | | | Fatuous.† Congenital. | | Fatuous.† Subsequent to Birth. | | Fatuous.† Congenital and Acquired. | | | Furious and Fatuous. | | | Population. | | | Proportion of Insane to Population. |
|---|---|---|---|---|---|---|---|---|---|---|---|---|---|---|---|---|---|
| | M. | F. | Total. | M. | F. | M. | F. | M. | F. | Total. | M. | F. | Total. | Males. | Females. | Total. | |
| Arendal ......... | 1 | ... | 1 | ... | ... | 1 | 8 | 1 | 8 | 9 | 2 | 8 | 10 | 1,645 | 1,917 | 3,562 | 1:356·2 |
| Grimstad ...... | ... | ... | ... | ... | ... | ... | 1 | ... | 1 | 1 | ... | 1 | 1 | 379 | 427 | 806 | 1:806 |
| Lillesand ...... | ... | ... | ... | ... | ... | 1 | ... | 1 | ... | 1 | 1 | ... | 1 | 291 | 280 | 571 | 1:571 |
| Christianssand | 1 | 9 | 10 | 2 | 2 | 4 | 2 | 6 | 4 | 10 | 7 | 13 | 20 | 3,962 | 4,387 | 8,349 | 1:447·4 |
| Mandal ......... | 1 | ... | 1 | 1 | 2 | 2 | ... | 3 | 2 | 5 | 4 | 2 | 6 | 1,041 | 1,263 | 2,304 | 1:384 |
| Farsund ......... | ... | ... | ... | 1 | 1 | 1 | 1 | 2 | 2 | 4 | 2 | 2 | 4 | 488 | 607 | 1,095 | 1:273·7 |
| Flekkefjord ... | 1 | ... | 1 | 1 | 1 | ... | ... | 1 | 1 | 2 | 2 | 1 | 3 | 705 | 905 | 1,610 | 1:536·6 |
| Soggendal ...... | ... | ... | ... | ... | ... | ... | ... | ... | ... | ... | ... | ... | ... | 167 | 181 | 348 | — |
| Egersund ...... | ... | ... | ... | 1 | ... | ... | 1 | 1 | 1 | 2 | 1 | 1 | 2 | 587 | 644 | 1,231 | 1:615·5 |
| Stavanger ...... | 4 | 1 | 5 | ... | 2 | 9 | 12 | 9 | 14 | 23 | 13 | 15 | 28 | 4,158 | 4,488 | 8,646 | 1:308·8 |
| Bergen ......... | 3 | 5 | 8 | 6 | 6 | 11 | 10 | 17 | 16 | 33 | 20 | 21 | 41 | 11,016 | 12,513 | 23,529 | 1:573·9 |
| Aalesund ...... | 1 | 1 | 2 | ... | ... | ... | ... | ... | ... | ... | 1 | 1 | 2 | 565 | 592 | 1,157 | 1:578·5 |
| Molde ............ | 2 | ... | 2 | ... | 2 | ... | ... | ... | 2 | 2 | 2 | 2 | 4 | 557 | 626 | 1,183 | 1:295·7 |
| Christianssund | ... | ... | ... | 1 | 1 | ... | ... | 1 | 1 | 2 | 1 | 1 | 2 | 1,498 | 1,665 | 3,163 | 1:1581·5 |
| Throndhjem ... | 24 | 10 | 34 | 6 | 3 | 6 | 4 | 12 | 7 | 19 | 36 | 17 | 53 | 6,954 | 7,824 | 14,778 | 1:278·8 |
| Levanger ...... | ... | ... | ... | ... | ... | ... | ... | ... | ... | ... | ... | ... | ... | 268 | 390 | 758 | — |
| Bodöe ............ | ... | ... | ... | ... | ... | ... | ... | ... | ... | ... | ... | ... | ... | 121 | 137 | 258 | — |
| Tromsöe ......... | 2 | ... | 2 | 1 | 1 | ... | 1 | 1 | 2 | 3 | 3 | 2 | 5 | 973 | 1,038 | 2,011 | 1:402·2 |
| Hammerfest ... | ... | ... | ... | 2 | ... | 1 | ... | 3 | ... | 3 | 3 | ... | 3 | 532 | 395 | 927 | 1:309 |
| Vardöe ......... | ... | ... | ... | ... | ... | ... | ... | ... | ... | ... | ... | ... | ... | 113 | 80 | 193 | — |
| Vadsöe ......... | ... | ... | ... | ... | ... | ... | 1 | ... | 1 | 1 | ... | 1 | 1 | 211 | 177 | 388 | 1:388 |
| Total ...... | 61 | 53 | 114 | 42 | 34 | 60 | 75 | 102 | 109 | 211 | 163 | 162 | 325 | 77,930 | 83,945 | 161,875 | 1:498·1 |

* The word used by Professor Holst is *Rasende* (Gale), which includes mania, monomania, and melancholia of Pinel and Esquirol. He defines the term to apply to those having a general disturbance or excitation of the cerebral functions, associated with violence, &c.

† The word used is *Fjanter*—cases marked by a weakness, or deficiency in the development, of the cerebral functions. He makes two subdivisions: congenital cases (idiocy), and acquired, or subsequent to birth, (dementia).

II.—*Table showing the Number of Insane in Country Districts, or Counties, in Norway, according to the Census of 31st of December,* 1845.

| Names of Districts. | Furious (Rasende). | | | Fatuous (Fjanter). Congenital. | | Fatuous (Fjanter). Subsequent to Birth. | | Fatuous (Fjanter). Congenital and Acquired. | | | Furious and Fatuous. | | | Population. | | | Proportion of Insane to Population. |
|---|---|---|---|---|---|---|---|---|---|---|---|---|---|---|---|---|---|
| | M. | F. | Total. | M. | F. | M. | F. | M. | F. | Total. | M. | F. | Total. | Males. | Females. | Total. | |
| Agershuus Amt* . . | 37 | 28 | 65 | 69 | 74 | 27 | 46 | 96 | 120 | 216 | 133 | 148 | 281 | 37,713 | 37,971 | 75,684 | 1:269·3 |
| Smaalehnenes . . . | 17 | 20 | 37 | 42 | 54 | 21 | 33 | 63 | 87 | 150 | 80 | 107 | 187 | 29,710 | 30,058 | 59,768 | 1:339·2 |
| Hedemarkens . . . . | 24 | 25 | 49 | 89 | 82 | 39 | 46 | 128 | 128 | 256 | 152 | 153 | 305 | 42,441 | 44,677 | 87,118 | 1:288·8 |
| Christians . . . . | 47 | 40 | 87 | 103 | 119 | 26 | 46 | 129 | 165 | 294 | 176 | 205 | 381 | 49,899 | 52,136 | 102,035 | 1:267·7 |
| Buskeruds . . . . | 30 | 44 | 74 | 81 | 76 | 32 | 62 | 113 | 138 | 251 | 143 | 182 | 325 | 34,984 | 36,129 | 71,113 | 1:218·8 |
| Jarlsbergs and Laurvigs | 22 | 21 | 43 | 44 | 34 | 22 | 34 | 66 | 68 | 134 | 88 | 89 | 177 | 26,074 | 26,592 | 52,666 | 1:296·9 |
| Bratsbergs . . . . | 36 | 36 | 72 | 48 | 42 | 27 | 33 | 75 | 75 | 150 | 111 | 111 | 222 | 30,148 | 31,711 | 61,859 | 1:278·6 |
| Nedenæs and Raabygdelaugets . . . . | 19 | 28 | 47 | 63 | 53 | 24 | 19 | 87 | 72 | 159 | 106 | 100 | 206 | 23,078 | 23,445 | 46,523 | 1:225·8 |
| Listers and Mandals . | 26 | 39 | 65 | 55 | 43 | 38 | 51 | 93 | 94 | 187 | 119 | 133 | 252 | 23,803 | 24,757 | 48,560 | 1:192·7 |
| Stavangers . . . . | 32 | 24 | 56 | 59 | 46 | 28 | 36 | 87 | 82 | 169 | 119 | 106 | 225 | 33,884 | 34,101 | 67,985 | 1:302·1 |
| South Bergenhuus . . | 41 | 33 | 74 | 68 | 61 | 33 | 43 | 101 | 104 | 205 | 142 | 137 | 279 | 46,012 | 47,448 | 93,460 | 1:335 |
| North Bergenhuus . . | 18 | 30 | 48 | 60 | 63 | 17 | 43 | 77 | 106 | 183 | 95 | 136 | 231 | 38,176 | 39,802 | 77,978 | 1:337·5 |
| Romsdals . . . . . | 20 | 27 | 47 | 49 | 46 | 25 | 30 | 74 | 76 | 150 | 94 | 103 | 197 | 37,240 | 38,571 | 70,811 | 1:384·9 |
| South Throndhjems . | 20 | 24 | 44 | 52 | 67 | 40 | 27 | 92 | 94 | 186 | 112 | 118 | 230 | 36,765 | 37,786 | 75,551 | 1:324·1 |
| North Throndhjems . | 16 | 25 | 41 | 58 | 69 | 25 | 31 | 83 | 100 | 183 | 99 | 125 | 224 | 32,267 | 33,545 | 65,812 | 1:293·8 |
| Nordlands . . . . | 14 | 12 | 26 | 43 | 42 | 26 | 24 | 69 | 66 | 135 | 83 | 78 | 161 | 31,974 | 33,280 | 65,254 | 1:405·3 |
| Finmarkens . . . . | 8 | 6 | 14 | 14 | 19 | 12 | 23 | 26 | 42 | 68 | 34 | 48 | 82 | 20,142 | 20,277 | 40,412 | 1:492·9 |
| Total . . . | 427 | 462 | 889 | 997 | 990 | 462 | 627 | 1459 | 1617 | 3076 | 1886 | 2079 | 3965 | 574,310 | 592,286 | 1,166,596 | 1:294·2 |
| Towns . . . . . . | 61 | 53 | 114 | 42 | 34 | 60 | 75 | 102 | 109 | 211 | 163 | 162 | 325 | 77,930 | 83,945 | 161,875 | 1:498·1 |
| Whole kingdom . . | 488 | 515 | 1003 | 1039 | 1024 | 522 | 702 | 1561 | 1726 | 3287 | 2049 | 2241 | 4290 | 652,240 | 676,231 | 1,328,471 | 1:309·7 |

* Amt is the largest civil division of the country, and may be considered equivalent to our county. Each Amt is presided over by a Government or Revenue Officer, who may be regarded as similar to our Sheriff.

III.—*The following Table shows the Number of the Insane in the aggregate Towns of Norway, during the Years* 1825–6, 1835, *and* 1845, *respectively.*

| | | 1845. | | | 1835. | | | 1825–6. | | |
|---|---|---|---|---|---|---|---|---|---|---|
| | | M. | F. | Total. | M. | F. | Total. | M. | F. | Total. |
| 1. Furious (Rasende) | Mania | 61 | 63 | 114 | 57 | 61 | 118 | 36 | 31 | 67 |
| | Monomania | | | | 35 | 45 | 80 | 34 | 31 | 65 |
| 2. Fatuous (Fjanter) | Subsequent to birth (dementia) | 60 | 75 | 135 | 35 | 26 | 61 | 31 | 23 | 54 |
| | From birth (idiocy) | 42 | 34 | 76 | 49 | 34 | 83 | 20 | 23 | 43 |
| Total | | 163 | 162 | 325 | 176 | 166 | 342 | 121 | 108 | 229 |

IV.—*The following Table shows the Number of the Insane in the aggregate Country Districts of Norway, during the Years* 1825–6, 1835, *and* 1845, *respectively.*

| | | 1845. | | | 1835. | | | 1825–6. | | |
|---|---|---|---|---|---|---|---|---|---|---|
| | | M. | F. | Total. | M. | F. | Total. | M. | F. | Total. |
| 1. Furious (Rasende) | Mania | 427 | 462 | 889 | 306 | 299 | 605 | 234 | 211 | 445 |
| | Monomania | | | | 269 | 286 | 555 | 164 | 147 | 311 |
| 2. Fatuous (Fjanter) | Subsequent to birth (dementia) | 462 | 627 | 1089 | 226 | 233 | 459 | 137 | 150 | 287 |
| | From birth (idiocy) | 997 | 990 | 1987 | 836 | 779 | 1615 | 349 | 288 | 637 |
| Total | | 1886 | 2079 | 3965 | 1637 | 1597 | 3234 | 884 | 796 | 1680 |

V.—*The following Table is made up of the two previous ones, and exhibits the Number of the Insane in the aggregate Towns and Country Districts of Norway—that is, in the whole Kingdom—during the Years* 1825–6, 1835, *and* 1845, *respectively.*

| | | 1845. | | | 1835. | | | 1825–6. | | |
|---|---|---|---|---|---|---|---|---|---|---|
| | | M. | F. | Total. | M. | F. | Total. | M. | F. | Total. |
| 1. Furious (Rasende) | Mania | 488 | 515 | 1003 | 363 | 360 | 723 | 270 | 242 | 512 |
| | Monomania | | | | 304 | 331 | 635 | 198 | 178 | 376 |
| 2. Fatuous (Fjanter) | Subsequent to birth (dementia) | 522 | 702 | 1224 | 261 | 259 | 520 | 168 | 173 | 341 |
| | From birth (idiocy) | 1039 | 1024 | 2062 | 885 | 813 | 1698 | 369 | 311 | 680 |
| Total | | 2049 | 2241 | 4290 | 1813 | 1763 | 3576 | 1005 | 904 | 1909 |

VI.—*Table showing the proportion of the Insane to the General Population of Norway, during the Years* 1825–6, 1835, *and* 1845, *respectively.*

| | 1845. | | | 1835. | | | 1825–6. | | |
|---|---|---|---|---|---|---|---|---|---|
| | M. | F. | Total. | M. | F. | Total. | M. | F. | Total. |
| 1. In towns . . . . | 1:478·1 | 1:518·2 | 1:498·1 | 1:349·2 | 1:406·9 | 1:377·2 | 1:449·4 | 1:553·9 | 1:498·7 |
| 2. In country districts | 1:304·5 | 1:284·9 | 1:294·2 | 1:320 | 1:339·3 | 1:329·6 | 1:516·6 | 1:603·6 | 1:557·8 |
| 3. Whole kingdom . | 1:318·9 | 1:301·3 | 1:309·7 | 1:322·9 | 1:345·7 | 1:334·1 | 1:508·5 | 1:597·5 | 1:550·7 |

VII.—There is a remarkable change in the number of insane during the two decennial periods from 1825 to 1835, and from 1835 to 1845, as the subjoined Table shows:—

| — | 1835-45. | | | 1825-35. | | |
|---|---|---|---|---|---|---|
| | Increased or diminished absolutely. | Proportion per cent. of increase or diminution. Without relation to the Population. | Proportion per cent. of increase or diminution. In proportion to the Population. | Increased absolutely. | Per cent. increased. Without relation to the Population. | Per cent. increased. In proportion to the Population. |
| 1. In towns . . . . | − 17 | − 5 % | −24·3% | + 113 | +49·3% | +37·9% |
| 2. In country districts . | +731 | +22·6– | +12 – | +1554 | +92·5– | +69 – |
| 3. Whole kingdom . . | +714 | +19·9– | + 7·9– | +1667 | +87·3– | +64·8– |

The number has, on the whole, increased during both decennial periods; but there is a diminution in the proportion of cases from towns.

VIII.—The increase has been greater during both decennial periods than the relative increase of the population:—

| | In 1835-45. | | In 1825-35. |
|---|---|---|---|
| In towns the increase has been . . . . | 25·5% | ...... | 11·7% |
| In country districts . . . . . . . . | 9·5– | ...... | 13·9– |
| In whole kingdom . . . . . . . . . | 11·2– | ...... | 13·6– |

Whereas during the first decennium an increase occurred among all classes of the insane, during the second it has been in the classes of dementia and idiocy; the number of cases of mania and melancholia (*rasende*) having considerably diminished, thus:—

| | | In 1835-45. | In 1825-35. |
|---|---|---|---|
| 1. Furious (Rasende) | Mania . . . / Melancholia . | − 26·1% | + 41% / + 69– |
| 2. Fatuous (Fjanter) | Dementia . . | + 135·4– | + 52– |
| | Idiocy . . . | + 21·5– | + 150– |

IX.—It will be noticed, from the foregoing Tables, that there is a much smaller proportion of insane in the towns than in the country, being in the former 1:498·1, and in the latter 1:294·2; hence it might be supposed that the causes of insanity are more rife in the latter. In Hölen, Sarpsborg, Lillehammer, Holmestrand, Aasgaardstrand, Soggendal, Levanger, Bodöe, and Vardöe, which are all thinly-peopled seaport or market towns,—Sarpsborg and Holmestrand, however, having respectively upwards of 1300 and 1700 inhabitants,—and which have an aggregate population of 5928, there are no insane persons. In the country districts, or *amts*, the number was greatest in Christians, Listers and Mandals, Buskeruds, Nedenæs and Raabygdelaugets amts—being respectively 1:137·1, 1:192·7, 1:218·8, and 1:225·8; least in Nordlands and Finmarkens amts—being respectively 1:405·3 and 1:493.

From these Tables it would appear that the insane population of Norway is about 5000. In 1825-6, it was 1909; in 1835, 3576; and in 1845, 4290. At this rate of increase, 5000 is not too large a number to set down against the year 1857, a period of twelve years having elapsed since the date of the latest census upon which my calculations are based. In truth, it must be far below the real number, but it is sufficiently accurate for my present purpose. We have already seen, on the other hand, that Gaustad has present accommodation for between 160 and 170 patients, and prospective accommodation for 250—say 250; and that the Mangelsgaarden Asylum cannot admit above 60. The other Norwegian asylums are—one in Opslö, a suburb, or part of Christiania, with room for 30 or 40 patients, in round numbers; one in Bergen, for 70 or 80; one in Throndjhem, for 60 or 70; one in Stavanger, for 8 or 10—all on the west coast—and one in Christiansand, for 30 or 40.* Now, if we take the maximum numbers given above as representing the actual accommodation provided at present in Norway for her insane population, we will find the total to be 550, which, being deducted from 5000, the supposed total number of insane persons in Norway at the present date, leaves the enormous balance of 4450 apparently inadequately provided for, or not provided for at all! Much, therefore, still remains to be achieved in Norway in regard to proper provision for her insane; but with her liberality, and modern views on the treatment of insanity, and the construction and management of asylums, I have no fear as to the ultimate result.

---

### Art. IV.—CHARLOTTE BRONTË—A PSYCHOLOGICAL STUDY.

* My informant was Professor Holst, if I remember aright: the numbers are merely approximative, and not actual numbers.

